# YZL-51N functions as a selective inhibitor of SIRT7 by NAD^+^ competition to impede DNA damage repair

**DOI:** 10.1016/j.isci.2024.110014

**Published:** 2024-05-16

**Authors:** Tian-Shu Kang, Yong-Ming Yan, Yuan Tian, Jun Zhang, Minghui Zhang, Yuxin Shu, Jinbo Huang, Jing He, Cheng-Tian Tao, Qian Zhu, Jinke Gu, Xiaopeng Lu, Yong-Xian Cheng, Wei-Guo Zhu

**Affiliations:** 1 International Cancer Center, Department of Biochemistry and Molecular Biology, Institute for Inheritance-Based Innovation of Chinese Medicine, School of Pharmaceutical Sciences, Shenzhen University Medical School, Shenzhen 518055, China; 2 Department Guangdong Key Laboratory of Genome Instability and Human Disease Prevention, Marshall Laboratory of Biomedical Engineering, Shenzhen University Medical School, Shenzhen 518055, China

**Keywords:** Pharmacology, Small molecule, Natural product biochemistry, Molecular biology experimental approach

## Abstract

The NAD^+^-dependent deacetylase SIRT7 is a pivotal regulator of DNA damage response (DDR) and a promising drug target for developing cancer therapeutics. However, limited progress has been made in SIRT7 modulator discovery. Here, we applied peptide-based deacetylase platforms for SIRT7 enzymatic evaluation and successfully identified a potent SIRT7 inhibitor **YZL-51N**. We initially isolated bioactive **YZL-51N** from cockroach (*Periplaneta americana*) extracts and then developed the *de novo* synthesis of this compound*.* Further investigation revealed that **YZL-51N** impaired SIRT7 enzymatic activities through occupation of the NAD^+^ binding pocket. **YZL-51N** attenuated DNA damage repair induced by ionizing radiation (IR) in colorectal cancer cells and exhibited a synergistic anticancer effect when used in combination with etoposide. Overall, our study not only identified **YZL-51N** as a selective SIRT7 inhibitor from insect resources, but also confirmed its potential use in combined chemo-radiotherapy by interfering in the DNA damage repair process.

## Highlights

• **YZL-51N** isolated from *P. americana* extracts is a potent SIRT7 inhibitor
• *De novo* synthesis of **YZL-51N** is accomplished
• **YZL-51N** functions as a multiple enzymatic inhibitor of SIRT7
• **YZL-51N** attenuates DNA damage repair and suppresses cancer cell survival

## Graphical abstract

**Figure:**
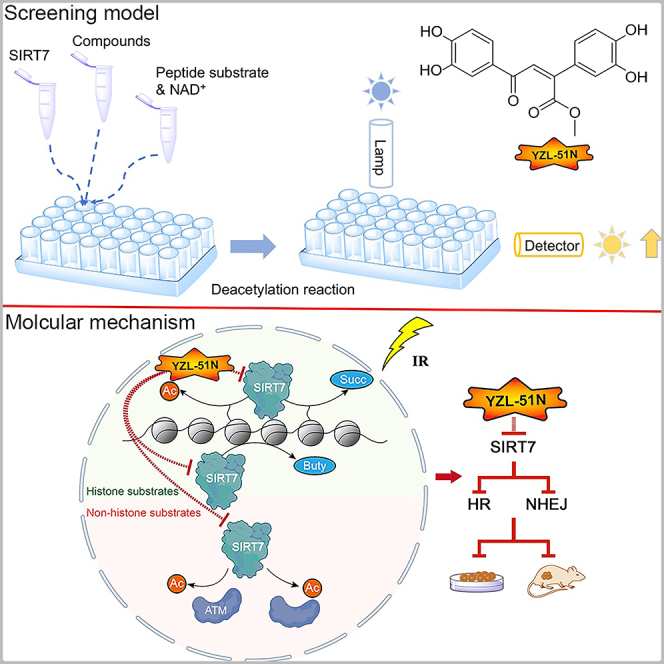

## Introduction

Mounting evidence indicates that endogenous metabolites and exposure to environmental factors can induce multiple types of DNA damage, contributing to genome instability and eventually accelerating tumorigenesis.^1^^,^^2^ Epigenetic modifiers are important regulators of chromatin remodeling as well as recruitment of repair factors that facilitate DNA damage repair.^3^ Thus, the development of therapeutic molecules targeting these modifiers is of broad interest for their potential use in cancer treatment, either alone or in combined chemoradiotherapy.

Silent Information Regulator 2 (SIR2) homologs are a family of NAD^+^-dependent histone deacetylases (HDACs) that exist in a wide range of species ranging from bacteria to humans.^4^ As a mammalian SIR2 homolog, SIRT7 is increasingly studied and has been identified as an important regulator of various diseases. Dysfunction in SIRT7 activity can lead to partial embryonic lethality, shortened life span, fatty liver, kyphosis and premature aging.^5^^,^^6^ SIRT7 was primarily identified as a selective H3K18ac deacetylase regulating cellular transformation and tumorigenesis.^7^ In addition, SIRT7 overexpression was detected in a wide variety of malignancies, including colorectal cancer, pancreatic cancer, and breast cancer.^8^^,^^9^^,^^10^^,^^11^ In the nucleolus, SIRT7 not only maintains rDNA stability, but also participates in pre-rRNA processing and rRNA maturation.^12^^,^^13^ In recent years, SIRT7 was revealed as an important regulator of DNA damage repair and a promising drug target in cancer treatment. When DNA double-strand breaks (DSBs) occur, SIRT7 binds to DSBs in a PARP1-dependent manner, modulating H3K18ac deacetylation for 53BP1 recruitment and H3K122succ desuccinylation for chromatin condensation.^14^^,^^15^ In addition, SIRT7-mediated ataxia telangiectasia mutated (ATM) deacetylation is a prerequisite for ATM dephosphorylation and inactivation during the late stage of DDR.^16^ Importantly, decreased SIRT7 levels after chemo-radiotherapy correlated well with improved therapeutic effect in patients with rectal cancer, implicating SIRT7 intervention as a potential clinical strategy to increase the effectiveness of chemo-radiotherapy in cancer patients.^17^

Several natural compounds extracted from fungi, marine life, and plants have been identified as histone deacetylase (HDAC) inhibitors. For example, trichostatin A (TSA), the first natural HDAC inhibitor, is derived from *Streptomyces hygroscopicus* and selectively inhibits class I and II HDACs.^18^^,^^19^ In addition, romidepsin (FK228), produced from *Chromobacterium violaceum*, selectively inhibits HDAC1/2 over HDAC4/6. Romidepsin has been approved by the United States Food and Drug Administration for treatment of cutaneous T cell lymphoma (CTCL) and peripheral T cell lymphoma (PTCL).^20^^,^^21^ Regarding class III HDACs (sirtuins), some representative inhibitors have been identified for SIRT1-6, such as EX527,^22^^,^^23^ AGK2,^24^ 3-HYP,^25^ suramin,^26^^,^^27^ H3K9TSu peptide,^28^ masked tetrazole compound,^29^ OSS_128167,^30^ and catechin gallate.^31^ For a long time, the NAD^+^ hydrolysis product nicotinamide (NAM) was used to inhibit SIRT7 activity, although it is in fact a pan-sirtuins inhibitor.^32^ Now, SIRT7 inhibitor identification is drawing increasing attention, and a few small molecules were considerably to reduce SIRT7 activity.^33^^,^^34^^,^^35^ Considering the limited progress in SIRT7 inhibitors development especially for the natural compounds, we were interested to systematically find such bioactive molecules, explore the synthesis technology and investigate chemical intervention on tumor models.

Cockroaches, which have existed for millions of years on earth,^36^ are associated with some common conditions, such as allergy and asthma.^37^ However, several bioactive molecules derived from cockroaches have shown considerable medicinal utility. For example, chitosan, which was isolated from the cockroach exoskeleton, showed antimicrobial activity against both Gram-positive and Gram-negative bacteria.^38^ Periplanetasin-2, which was derived from cockroach peptide extracts, has been shown to exert anti-fungal activity against *Candida albicans* by inducing oxidative stress and increasing reactive oxygen species (ROS) production in the mitochondria.^39^ In addition, some cockroach-derived small molecules with a ten-membered macrolactam or a morpholine motif also showed anti-angiogenic activity.^40^ Moreover, Kangfuxin liquid, the crude ethanol extract of *P. americana* and a marketed drug in China has achieved beneficial clinical treatment for gastric ulcers, burns and scalds.^41^ Although the detailed molecular mechanisms of *P. americana* extracts for a range of disorders remain largely unknown, we consider that compounds from *P. americana* can target mammalian proteins and regulate cell signaling pathways. We were thus inspired to see whether some compounds from *P. americana* or other nature resources can inhibit SIRT7 deacetylase activity and have the potential for use in cancer treatment.

In this study, by screening our natural products library, **YZL-51N**, a previously undescribed nonpeptidal structure from *P. americana* extracts, was proved to be a potent and selective SIRT7 inhibitor. We demonstrated the synergistic effect of **YZL-51N** in combined chemo-radiotherapy by interfering SIRT7-mediated DNA damage repair in colorectal cancer cells. Our discovery of **YZL-51N** brings a new insight into natural insect resources and guides further derivatives optimization to deal with cancer.

## Results

### *P. americana* extract YZL-51N is an identified SIRT7 inhibitor

**Figure 1:**
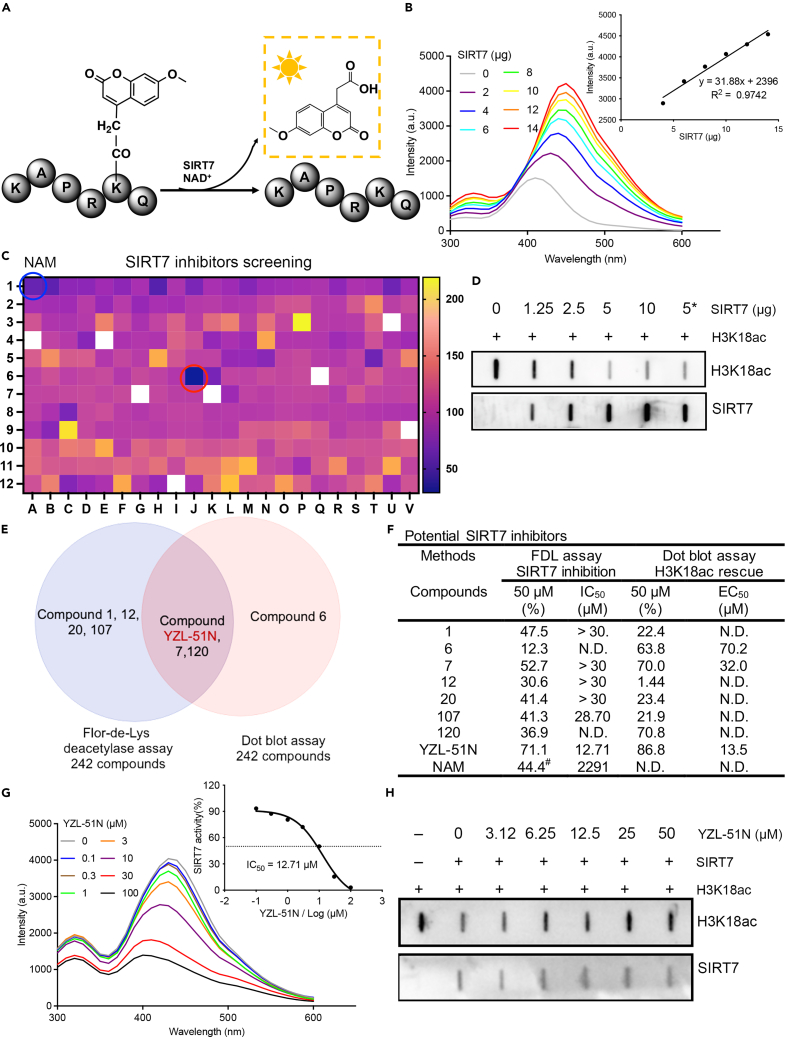
*P. americana* extract YZL-51N is an identified SIRT7 inhibitor (A) Schematic representation showed peptide-based fluorescent probe for SIRT7 activity detection. (B) Fluorescence intensity emission spectra indicated the enzymatic activities of 0–14 μg SIRT7 with 10 μM peptide substrates and 0.5 mM NAD^+^ at 37°C for 90 min. Insert: Linear relationship between the luminescence intensity (λ = 450 nm) and SIRT7 concentrations. (C) Heatmap comparing the inhibitory effects of 242 natural products on SIRT7 deacetylation activity by FDL assays. Notes: Blanks indicate not determined (N.D.). Blue circle indicates the pan-sirtuin inhibitor (NAM). Red circle indicates compound **YZL-51N**. (D) The reaction system containing 0–10 μg SIRT7, 0.5 mM NAD^+^, and 10 μM H3K18ac peptides was incubated at 37°C for 90 min. Dot blot was performed to show serial dilutions of purified SIRT7-mediated H3K18 deacetylation. PC, active SIRT7 as positive control purchased from Bio Vision Inc. (E) Venn diagram showing the SIRT7 inhibitor candidates identified in FDL assays and Dot blot assays. (F) List of potential SIRT7 inhibitors identified in two rounds of screening. ^**#**^, 2 mM NAM treatment. (G) Fluorescence spectra showing **YZL-51N**-mediated signals reduction in the presence of SIRT7, peptide substrate, and NAD^+^. Insert: Plots showing dose-dependent inhibitory effect of **YZL-51N** on SIRT7 activity. IC_50_ value: approximately 12.71 μM. (H) Dot blot assay showing the dose-dependent effect of **YZL-51N** on the inhibition of SIRT7-mediated H3K18ac deacetylation. See also Figures S1–S5.

To assess SIRT7 deacetylase activity using Fluor de Lys (FDL) assays, the peptide ARTKQTARKSTGGKAPRK∗QLAGGK was synthesized and characterized as a substrate using fluorophore 7-methoxycoumarin-4-acetic acid (∗, MCA) (Figures 1A, S1A, and S1B). In the presence of both SIRT7 and NAD^+^, a luminescence response was detected in the linear range (y = 31.88x + 2396, R^2^ = 0.9742) (Figure 1B). We screened our preserved library of 242 natural products to find potential SIRT7 inhibitors. According to the screening heatmap shown in Figure 1C, we successfully identified several compounds (**1**, **7**, **107**, **YZL-51N**, and **120**) as potential candidates, among which, *P. americana* extract **YZL-51N** showed the greatest inhibitory effect.

To avoid the bias of the fluorescence assay, we also performed dot blot assays for the effects of natural compounds on SIRT7-mediated deacetylation of H3K18ac peptides *in vitro* (Figures S2A and S2B). In this system, SIRT7 mediated a dose-dependent deacetylation of H3K18ac (Figure 1D). Dot blot screening results revealed that four of the compounds (**6**, **7**, **YZL-51N** and **120**) rescued H3K18 acetylation at the dose of 50 μM (Figure S3). Hence, both FDL assays and dot blot assays implicated compounds **7**, **YZL-51N**, **120** as potential SIRT7 inhibitors (Figure 1E). Based on comparisons of all the candidates, we selected **YZL-51N** as the best for further investigation (Figure 1F). In the FDL assay, we observed a dose-dependent inhibition of SIRT7 by compound **YZL-51N** (IC_50_ = 12.71 μM), along with much weaker inhibition of SIRT7 by NAM (IC_50_ = 2.291 mM) (Figures 1G and S4). In contrast, compounds **1**, **7**, **12**, **20**, **107** and **120** did not show a dose-dependent inhibition of SIRT7 (Figure S5). Dot blot assays also revealed a dose-dependent accumulation of H3K18ac by using **YZL-51N** (EC_50_ = 13.5 μM) (Figure 1H). Thus, *P. americana* derived **YZL-51N** was confirmed as an identified SIRT7 inhibitor using parallel peptide-based screening platforms.

### YZL-51N selectively inhibits SIRT7 deacetylase activity

**Figure 2:**
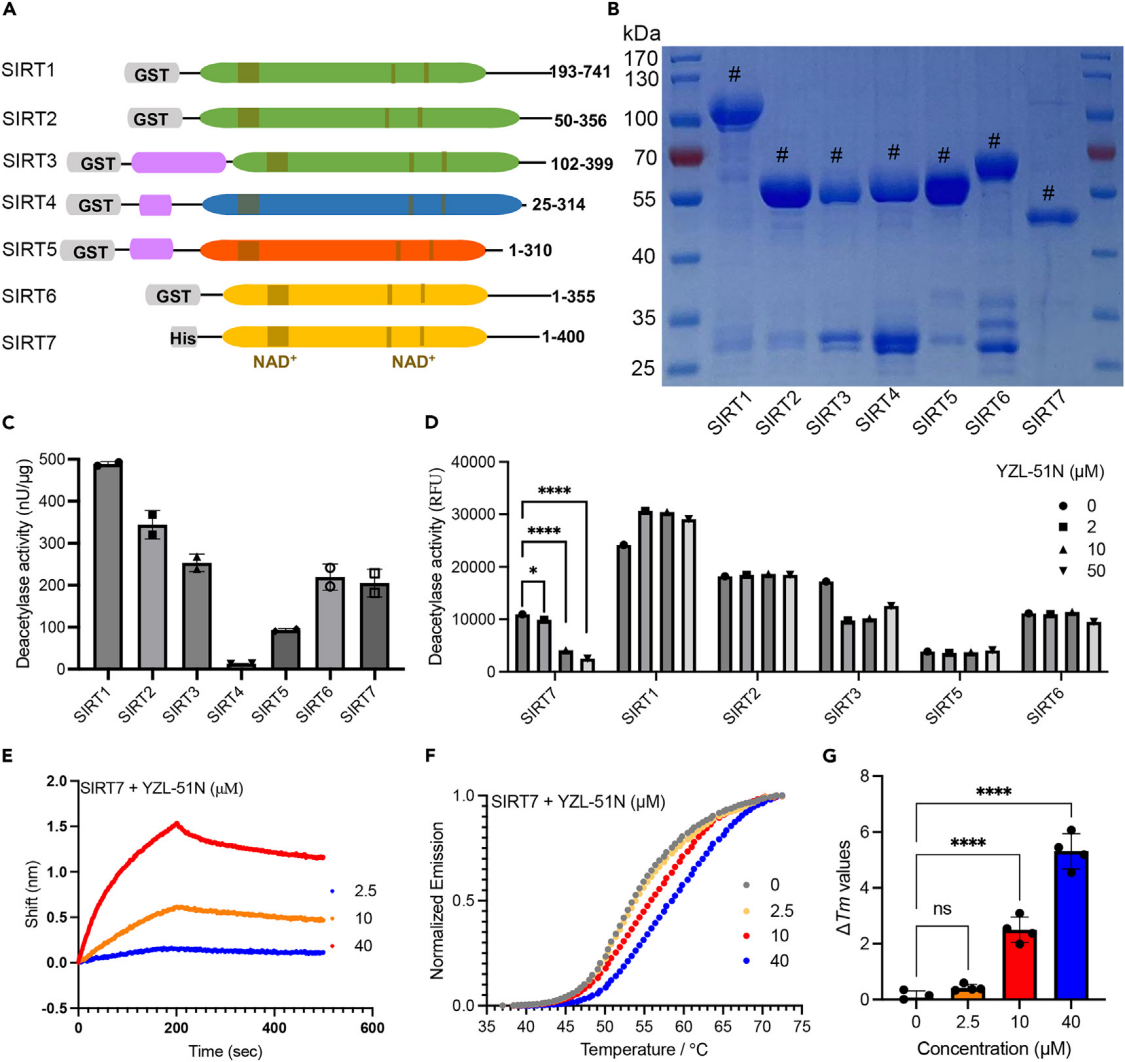
YZL-51N selectively inhibits SIRT7 deacetylase activity (A) Schematic representation of recombinant human sirtuins, including tags, NAD^+^ binding domain (gray), mitochondrial targeting domain (purple) and different subtypes (green, blue, yellow and red). (B) Coomassie brilliant blue staining showing the purified recombinant sirtuins. (C) Fluorometric ELISAs were performed to show the deacetylase activities of 10 μg recombinant sirtuins according to the manufacturer’s instruction. Data represent the mean of two independent experiments. (D) ELISAs were performed to show the inhibitory activities of **YZL-51N** at different concentrations on SIRT1/2/3/5/6/7. Data were presented as the mean ± SD of three independent experiments and statistical analyses were performed using one-way ANOVA. ∗*p* < 0.05; ∗∗∗∗*p* < 0.0001. (E) Biotinylated SIRT7 proteins were immobilized onto the surface of SSA biosensors. 0–40 μM **YZL-51N** were allowed to flow though the chips at room temperature in PBST buffer (pH 7.4). Kinetic parameters and affinities were calculated using Octet Data Analysis software version 7.0 (Fortebio). (F) Protein thermal shift assays were conducted to show the thermostability changes of SIRT7 with 0–40 μM **YZL-51N**. (G) Quantification of ΔTm values in (F). Data were presented as the mean ± SD of four independent experiments. Statistical analyses were performed with one-way ANOVA. ∗∗∗∗*p* < 0.0001; ns stands for no significant change. See also Figures S6 and S7.

To determine the selectivity of **YZL-51N**, we assessed its inhibitory activity against recombinant human sirtuins expressed in *E. coli* through deacetylation assays *in vitro* (Figures 2A and 2B). ELISAs revealed that all the purified sirtuins were enzymatically active, except for SIRT4, which has been reported to exhibit much weaker deacetylation activity compared with that of the other sirtuins (Figure 2C).^42^ Notably, unlike its effect on SIRT7, **YZL-51N** did not inhibit the deacetylase activities of other sirtuins in a dose-dependent manner (Figure 2D). We next validated the direct interaction between **YZL-51N** and SIRT1/2/6/7 using bio-layer interferometry (BLI) assays to compare their binding affinities. As shown in Figures 2E and S6A–S6C, we observed a potent interaction between **YZL-51N** and SIRT7 with a *K*_D_ of 3.3 μM, which was stronger than that of the interactions with SIRT1/2/6. To gain insights into the ability of **YZL-51N** to affect sirtuin thermostability, we conducted protein thermal shift assays. At a concentration of 10 μM, **YZL-51N** caused shifts in the melting curves for SIRT7, SIRT6 and SIRT1 of 2.86°C, 1.0°C and 0.45°C (Figures S7A–S7C). In addition, we observed a dose-dependent increase in the Δ*Tm* for SIRT7 following **YZL-51N** treatment (Figures 2F and 2G). Taken together, these findings confirm that **YZL-51N** is a selective inhibitor of SIRT7.

### *P. americana* extraction and *de novo* synthesis of YZL-51N

**Figure 3:**
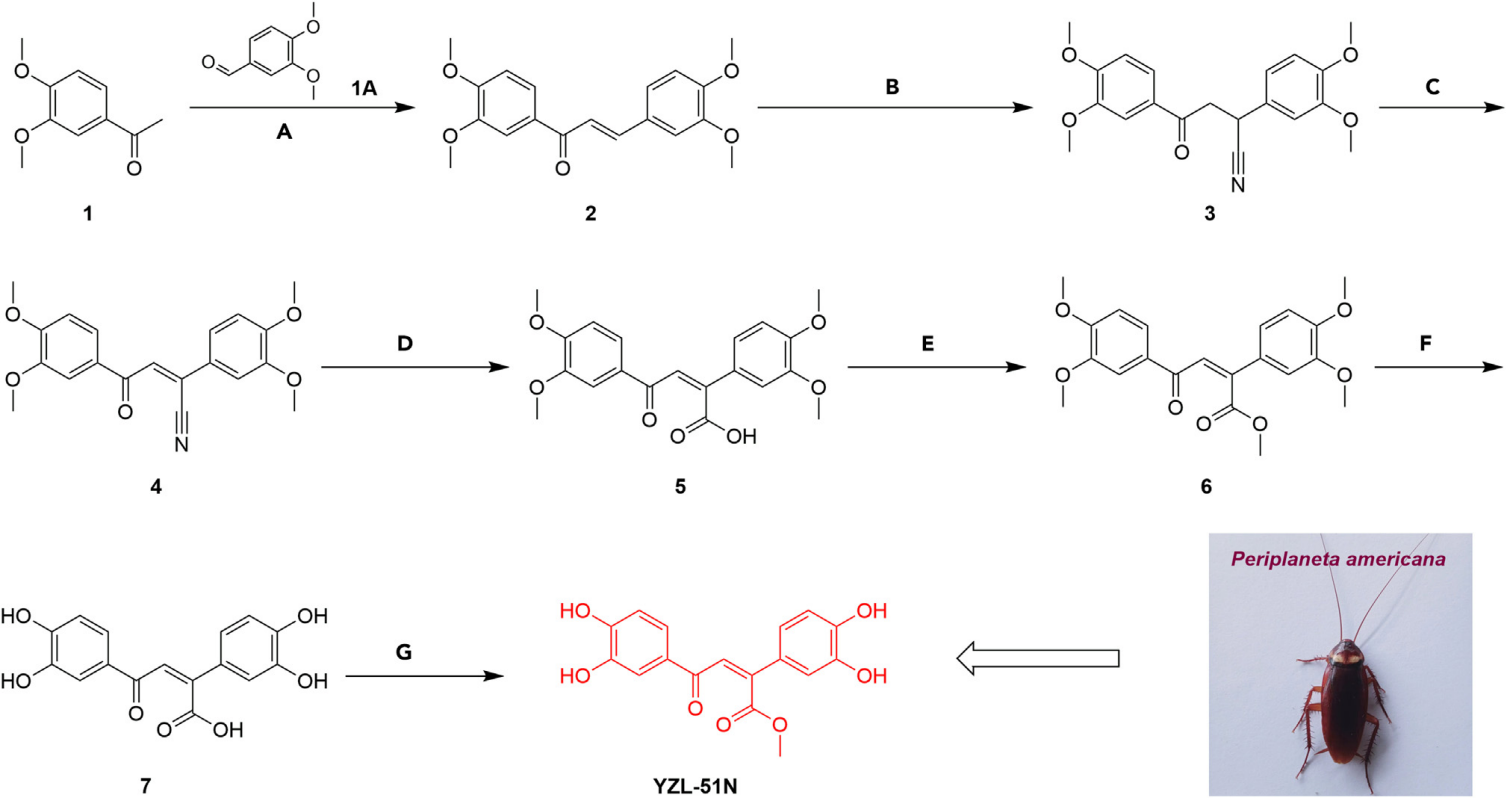
*De novo* synthesis of YZL-51N Reagents and conditions: (A) NaOH, MeOH, 60°C, 12 h, 89%; (B) Acetone, acetone cyanohydrin, N (CH_3_)_4_OH, 60°C 10 h, 91.4%; (C) Toluene, DDQ, N_2_, 120°C, 6 h, 51.1%; (D) MeOH, NaOH, 60°C, 2 h; (E) DMF, K_2_CO_3_, CH_3_I, N_2_, 20°C 6 h, 90%; (F) BBr3, DCM, 0°C, 4 h; (G) H_2_SO_4_, MeOH, 60°C, 2 h, 11.3%. See also Figures S15–S29 and Scheme S1.

The identified SIRT7 inhibitor **YZL-51N** was initially isolated from *P. americana* extracts. To obtain this compound, we used a combination of column chromatography. Eventually, we harvested approximately 1.5 mg compound **YZL-51N** from 30 kg dried *P. americana*. To achieve large-scale production of **YZL-51N**, the seven-step process for the *de novo* synthesis of **YZL-51N** was also designed using commercially available 3,4-dimethoxyacetophenone and veratraldehyde as the starting materials (Figure 3). The synthesized **YZL-51N** has been validated to be consistent with the isolated **YZL-51N** on the bases of HRESIMS, ^1^H NMR, ^13^C NMR, and DEPT spectra (Figures S15–S29 and Scheme S1).

### YZL-51N is a NAD^+^ competitive inhibitor of SIRT7

**Figure 4:**
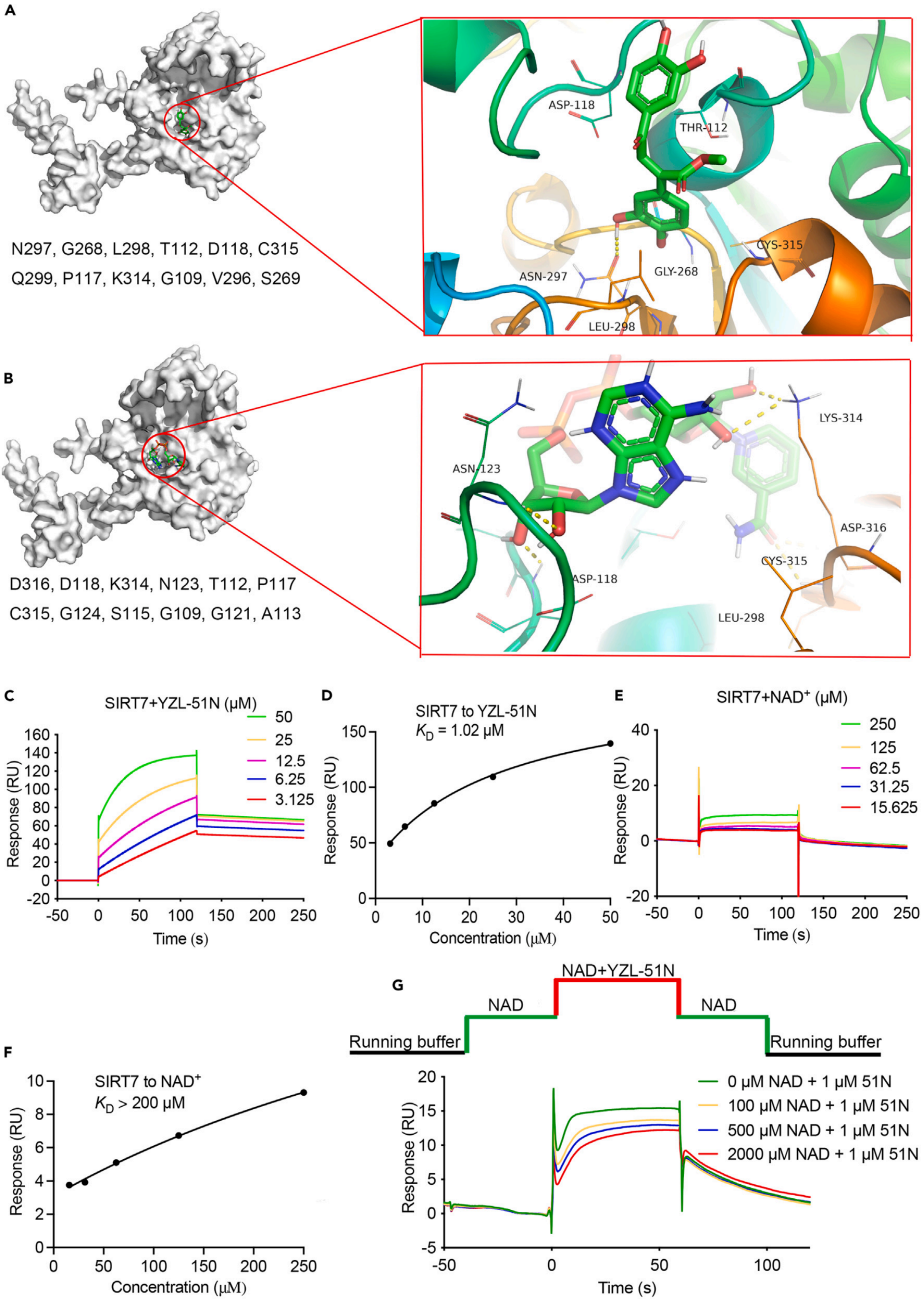
YZL-51N is a NAD^+^ competitive inhibitor of SIRT7 (A and B) Three-dimensional structures of ligand-protein docking revealed the binding pocket (Left) and main interacting residues (Right) in SIRT7 of **YZL-51N** (A) and NAD^+^ (B). (C–F) SPR assays were performed to indicate the binding affinity of SIRT7 with **YZL-51N** or NAD^+^. Recombinant SIRT7 proteins were immobilized on the chips. **YZL-51N** (3.125–50 μM) (C and D) or NAD^+^ (15.625–250 μM) (E and F) then passed through the chips and the response signals were collected. (G) **YZL-51N** competed with NAD^+^ to bind SIRT7 in SPR competitive assay using the “A-AB-A” model. See also Figures S8 and S9 and Table S1.

We next conducted molecular docking studies to gain further insights into the interaction of **YZL-51N** with SIRT7. According to the AlphaFold analysis (AF-Q9NRC8-F1),^43^^,^^44^ **YZL-51N** preferentially interacted with 12 amino acids in SIRT7 via multiple hydrogen bonding interactions (Figure 4A). Interestingly, the coenzyme NAD^+^ also interacted with a similar pocket in SIRT7, with six overlapped amino acids (Gly109, Thr112, Pro117, Asp118, Lys314 and Cys315) identified in the interactions (Figure 4B). To further validate whether these residues are responsible for the interaction between **YZL-51N** and SIRT7, we mutated six interacted amino acids and performed BLI assays to detect the binding affinity changes (Table S1). As shown in Figure S8A, several mutant proteins exhibited remarkable decreased binding signals as compared with the WT SIRT7. In addition, we conducted fluorescent ELISA tests to detect the discrepancies in SIRT7 deacetylase activities. D118A, N297A, L298A and C315A mutations demonstrated downregulated deacetylase activities as compared with the WT SIRT7 (Figure S8B). Among all sirtuin members, SIRT1, SIRT6 and SIRT7 are the three members mainly located in the nucleus in mammalian cells. We thus conducted additional docking experiments for **YZL-51N** and NAD^+^ with SIRT1 or SIRT6. Interestingly, **YZL-51N** and NAD^+^ interacted with SIRT1 or SIRT6 by different amino acids, which may possibly lead to intermolecular force differences and explain the selectivity of **YZL-51N** toward SIRT7 (Figure S9).

To further elucidate the mechanism by which **YZL-51N** inhibits SIRT7, we analyzed the kinetics of **YZL-51N** and NAD^+^ binding to SIRT7 using surface plasmon resonance (SPR) assays. The data revealed that **YZL-51N** bound to SIRT7 with a *K*_D_ = 1.02 μM, an association rate (*k*_on_) of 618.5 1/Ms and a disassociation rate (*k*_off_) of 6.315 × 10^−4^ 1/s (Figures 4C and 4D). We noticed that NAD^+^ bound to SIRT7 with a much weaker affinity (200-fold; *K*_D_ >200 μM) than **YZL-51N** (Figures 4E and 4F). To confirm the possibility of competitive binding of **YZL-51N** with NAD^+^ to SIRT7, we performed SPR competition assays using an A-AB-A injection model. In this model, NAD^+^ was priorly used at a series of concentration to bind SIRT7, and then followed by the **YZL-51N** competition. We observed that the binding signals of NAD^+^ to SIRT7 decreased in the presence of **YZL-51N**. In addition, the competitive binding was even observed in the presence of 2 mM of NAD^+^ (approximately 10-fold *K*_D_ concentration)(Figure 4G). Together, these data indicated that **YZL-51N** is a NAD^+^ competitive inhibitor of SIRT7.

### YZL-51N inhibits SIRT7 enzymatic activity in colon cancer cells

**Figure 5:**
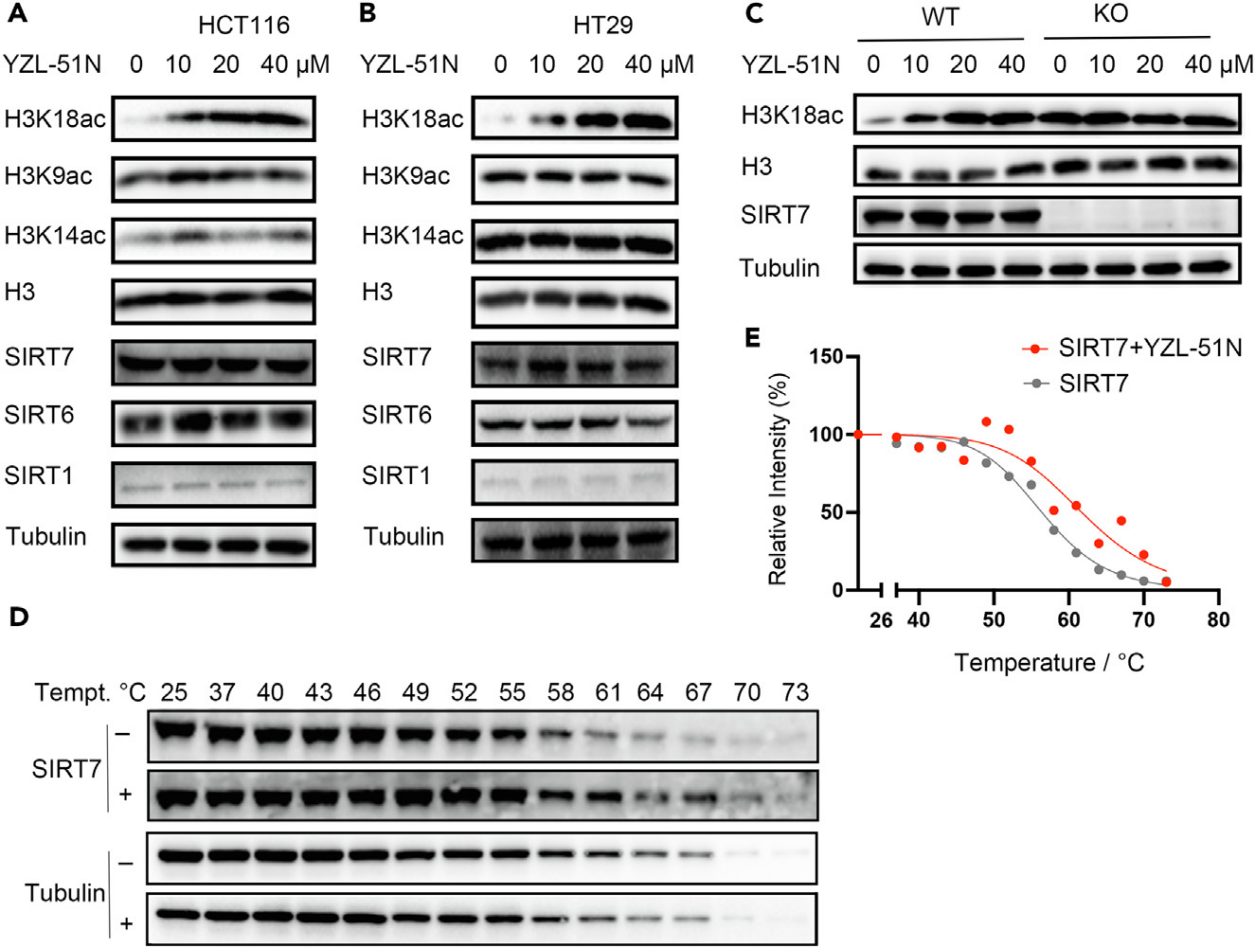
YZL-51N inhibits SIRT7 deacetylase activity in colon cancer cells (A) HCT116 cells were treated with 0–40 μM **YZL-51N** for 8 h. Whole cell lysates were extracted and probed with the indicated antibodies. (B) HT29 cells were treated with **YZL-51N** as described in (A). Whole cell lysates were extracted and monitored by using the indicated antibodies. (C) HCT116 WT and SIRT7 KO cells were treated with **YZL-51N.** Both cell lysates were extracted and probed with the indicated antibodies. (D) HCT116 cell lysates were incubated with/without 20 μM **YZL-51N** at a temperature range. The thermostability of SIRT7 and Tubulin were detected with western blot. (E) Quantification of western blots in (D). See also Figures S10 and S11.

To evaluate the inhibitory effect of **YZL-51N** on SIRT7 deacetylase activity at the cellular level, we treated colorectal cancer cells (HCT116 and HT29) with increasing concentrations of this compound. As shown in Figure 5A, the level of H3K18ac was increased in HCT116 cells treated with **YZL-51N** in a dose-dependent manner, whereas no marked changes were observed in the levels of H3K9ac and H3K14ac. Similar results were also observed in HT29 cells (Figure 5B). We also generated SIRT7 knockout (KO) HCT116 cells using CRISPR-Cas9 technology to compare the H3K18ac changes induced by **YZL-51N** treatment in both wild-type (WT) and SIRT7 KO cells. Unlike the WT cells, SIRT7 KO cells had high basal levels of H3K18ac, which were not further changed after **YZL-51N** treatment (Figure 5C). Previous studies suggested that SIRT7 has diverse enzymatic activities (debutyrylation,^45^ decrotonylation; ^46^ desuccinylation^15^ and auto-ADP-ribosylation^47^). We also detected a dose-dependent increase in H3K122succ, another histone substrate of SIRT7, after **YZL-51N** treatment (Figure S10A). Similar to the increases of H3K18ac and H3K122succ levels, **YZL-51N** treatment led to a marked pan-butyrylation change in location of histones (Figure S10B). However, the same treatment had no obvious effects on pan-crotonylation, pan-glutarylation and PARylation (Figures S10C–S10E).

Next, we performed cellular thermal shift assays to investigate the binding of **YZL-51N** to endogenous sirtuins in whole cell lysates. As shown in Figures 5D and 5E, **YZL-51N** increased the thermostability of SIRT7, causing a 5.2°C shift in the melting curve, whereas there was no obvious effect of **YZL-51N** on the melting curve of SIRT6 or SIRT1 (Figure S11). Overall, these findings suggested that **YZL-51N** specifically suppressed SIRT7 enzymatic activities in colorectal cancer cells.

### YZL-51N attenuates DNA damage repair

**Figure 6:**
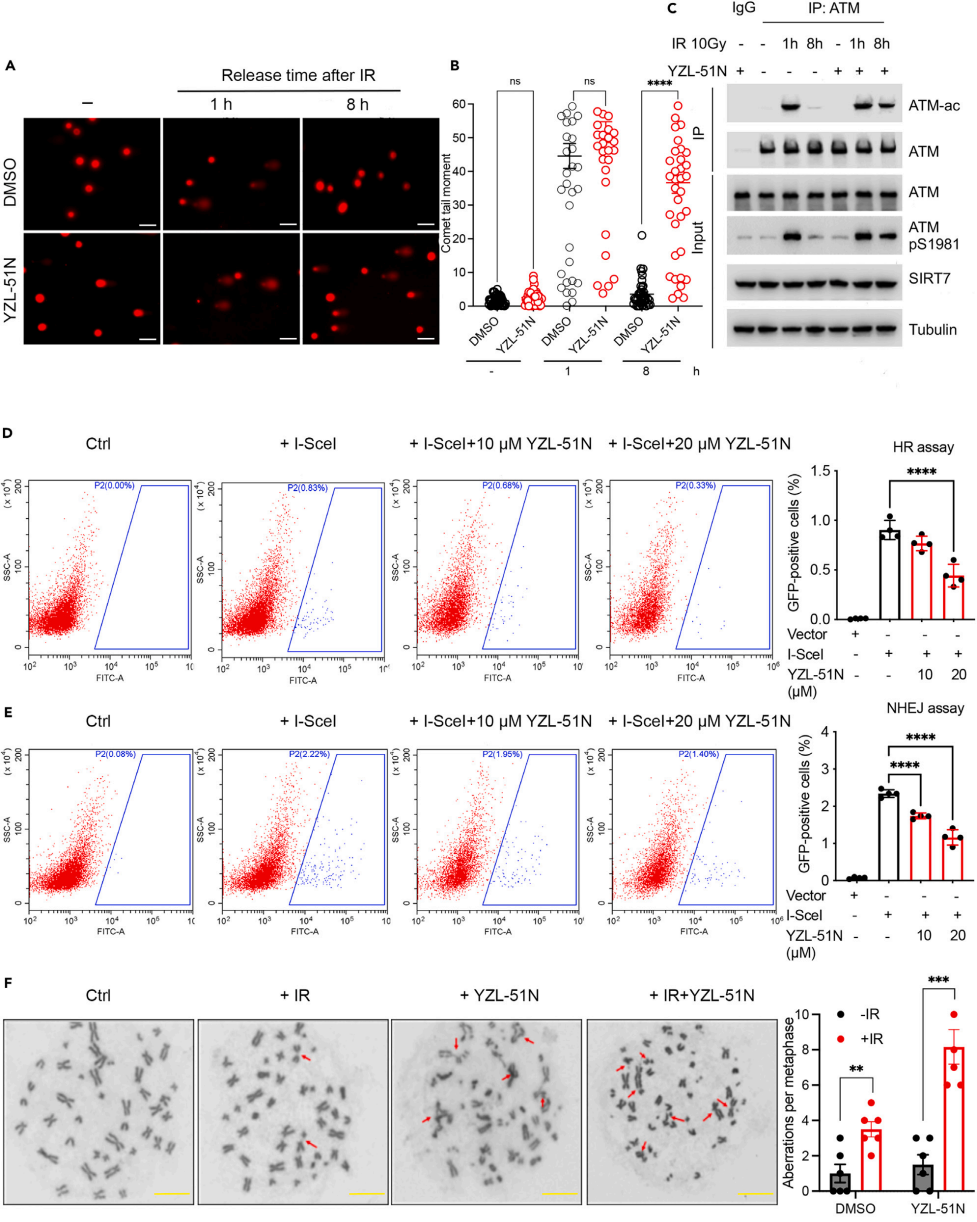
YZL-51N attenuates DNA damage repair (A and B) HCT116 cells were irradiated at 10 Gy and released for the indicated time periods with or without 20 μM **YZL-51N** treatment for comet assays. Representative images were shown in (A). Relative tail moments (>40 cells) were quantified for statistical analysis (B). Data were presented as the mean ± SD of three independent experiments. Statistical analyses were performed with Kolmogorov-Smirnov test. ∗∗∗∗*p* < 0.0001; ns stands for no significant change. Scale bar: 50 μm. (C) HCT116 cells were treated with IR at 10 Gy, and incubated with 20 μΜ **YZL-51N** for 2 h before cell collection for immunoprecipitation. (D and E) DR-HCT116 and EJ5-HCT116 cells were infected with I-SceI virus for 18 h, re-cultured for 2 days and then treated with **YZL-51N** for 12 h before cell collection for repair efficiency measurement. Representative flow cytometry images for HR (D) and NHEJ (E) efficiencies were shown. Data were presented as the mean ± SD of four independent experiments. Statistical analyses were performed with one-way ANOVA. ∗∗∗∗*p* < 0.0001. (F) HCT116 cells were irradiated at 4 Gy before treatment with or without 20 μM **YZL-51N** for 8 h. The cell samples were harvested 24 h later for metaphase spreading assay. Arrows indicate chromosome aberrations. Statistical analyses of aberrations were determined from six biological replicates in the right panel. Data were presented as the mean ± SD. Statistical analyses were performed with unpaired two-tailed Student’s t-test. ∗∗*p* < 0.01; ∗∗∗*p* < 0.001. Scale bar: 20 μm.

Considering the important roles of SIRT7 in DDR, we first evaluated the effect of **YZL-51N** on DNA damage repair using comet assays. After exposure to ionizing radiation (IR), long comet tails were observed in both **YZL-51N** treated and untreated cells, indicating comparable DNA damage. However, after 8 h of repair, **YZL-51N** treated cells showed much longer comet tails than the untreated cells, representing the delayed repair of damaged DNA (Figures 6A and 6B). Previously, we reported that SIRT7-mediated deacetylation of ATM during the late stage of DDR was a prerequisite for complete DNA damage repair.^16^ To evaluate the ability of **YZL-51N** to suppress this deacetylation, we performed immunoprecipitation assays to detect ATM deacetylation in HCT116 cells following the induction of DNA damage by exposure to IR. Without **YZL-51N** treatment, IR-induced acetylation and activation of ATM was recovered to almost basal levels by approximately 8 h post-irradiation. However, in the **YZL-51N** treated cells, high ATM acetylation and phosphorylation were still detectable at this time-point (Figure 6C), indicating that **YZL-51N** can also inhibit SIRT7-mediated deacetylation of non-histone substrates. DSBs are usually repaired by HR or NHEJ pathway. As shown in Figures 6D and 6E, flow cytometry data showed that **YZL-51N** treatment led to dose-dependent reductions of GFP signals in both DR-HCT116 and EJ5-HCT116 cells, indicating its ability to downregulate both HR and NHEJ repair. In addition, metaphase spreading assays also verified the increased chromatin instability induced by **YZL-51N** treatment after IR (Figure 6F).

### YZL-51N suppresses cancer cell survival

**Figure 7:**
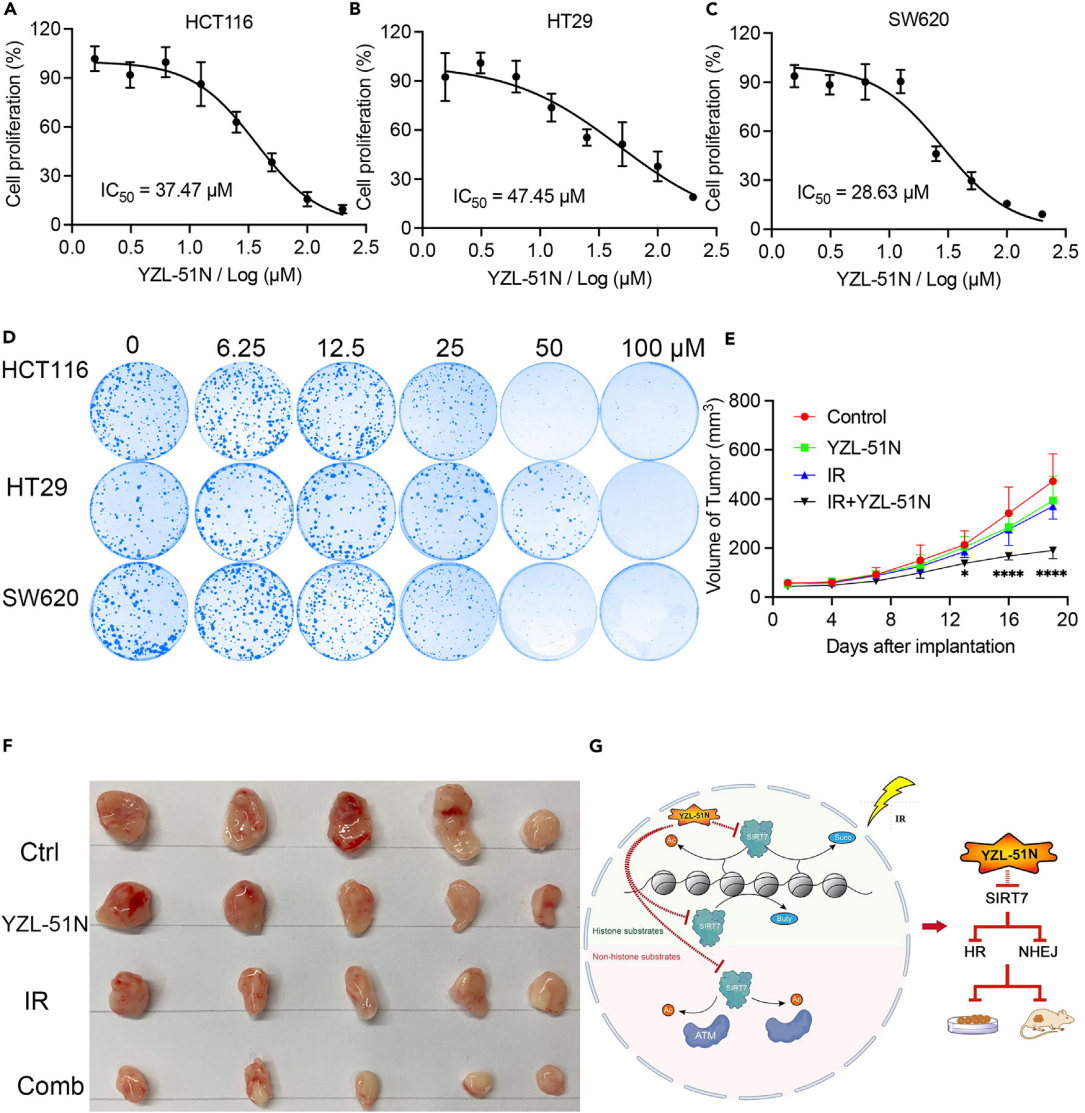
YZL-51N suppresses cancer cell survival (A–C) Cell proliferations of HCT116 (A), HT29 (B) and SW620 (C) were inhibited after **YZL-51N** treatment for 48 h. Consistent results were observed from four independent experiments. (D) HCT116, HT29 or SW620 cells were seeded into 6 well plates and treated with **YZL-51N** for 48 h. Cells were re-cultured in fresh medium for 14 days, fixed with 4% paraformaldehyde and stained with crystal violet for 1h. (E) BALB/c nude mice were injected subcutaneously with HCT116 cells. The control group was injected with PBS. **YZL-51N** treatment group was treated with 15 mg/kg **YZL-51N** every 3 days. IR treatment group was irradiated with 3 Gy every 3 days. The combination group was treated with 15 mg/kg **YZL-51N** and 3 Gy IR every 3 days. The tumor volume was monitored and used to plot the growth curve. *n* = 5 biological replicates for each group; Data were presented as the mean ± SD. Statistical analyses were performed with two-way ANOVA. ∗*p* < 0.05, combination group vs. control group; ∗∗∗∗*p* < 0.0001, combination group vs. control group. (F) Representative images of xenograft tumors. (G) Working model for **YZL-51N**-mediated SIRT7 inhibition and combined cancer therapy. See also Figures S12–S14 and Table S2.

Previous studies have suggested that knockdown of SIRT7 repressed the cell proliferation of colorectal carcinoma (CRC), which contains high expression of SIRT7.^48^ Having shown that **YZL-51N** can inhibit SIRT7 deacetylase activity and attenuate DNA damage repair, we were prompted to assess its potential for anti-tumor on colorectal cancers. As shown in Figures 7A–7C, high concentration of **YZL-51N** treatment decreased cell proliferations in three colorectal cancer cell lines (HCT116, HT29 and SW620). In addition, colony formation assays also revealed **YZL-51N** treatment caused a lower colony-forming efficiency (Figure 7D). Notably, different from the long-term drug treatment in CCK8 assays, we used low doses of **YZL-51N** and etoposide to treat cells for a short time in the drug combination experiment. Co-treatment with **YZL-51N** and etoposide induced a synergistic inhibition on cell proliferation (Figure S12 and Table S2). As shown in Figure S13, **YZL-51N** treatment made cells more sensitive to IR treatment. To determine whether **YZL-51N** has anti-tumor effect on *in vivo* system, we used HCT116 xenograft model and treated the mice with 15 mg/kg **YZL-51N** and 3 Gy IR, alone or combined. As shown in Figures 7E and 7F, either **YZL-51N** treatment or IR treatment led to a reduction on the tumor volume. In addition, co-treatment of **YZL-51N** and IR was more effective in the tumor therapy, which was consistent with the results observed in HCT116 cells. Additionally, we performed bioinformatical analysis for ADME (absorption, distribution, metabolism and excretion) properties of **YZL-51N** and got a systematic prediction. As shown in Figure S14, **YZL-51N** was predicted to have a good intestinal absorption and modest Caco-2 permeability. We also noticed that the water solubility and metabolic rate by cytochrome P450 of **YZL-51N** need to be improved in future drug optimization. Taken together, Figure 7G summarized the activities of **YZL-51N**-mediated SIRT7 inhibition and combined colon cancer therapy.

## Discussion

Emerging evidence suggests SIRT7 to be a promising drug target in cancer treatment. However, limited progress has been made in SIRT7 modulators discovery. In this study, we identified **YZL-51N**, a bioactive compound isolated from *P. americana* with previously unknown structure, as a selective SIRT7 inhibitor. We developed *de novo* synthesis of **YZL-51N** and revealed its pharmacological mechanism as competing with NAD^+^ to bind SIRT7. In response to DSBs, **YZL-51N** impaired DNA damage repair and increased the chromatin instability. Additionally, **YZL-51N** was confirmed to increase cancer cell sensitivity to IR and etoposide, suggesting its potential use in future cancer treatment.

Fluorescent peptide-based assay can be used to measure histone deacetylase activity and the impact of inhibitors as a high-throughput, quick and sensitive method. In this study, we adapted two parallel peptide-based screening platforms (FDL and dot blot) to own some advantages, such as stable dose-response relationship, high signal-to-noise ratio and double-checking validation. Eventually, **YZL-51N** was selected from both methods used to detect SIRT7 deacetylase activity, and proved efficient by a series of biochemical experiments. Our parallel peptide-based screening platforms are considered effective in future drug screening to identify other enzyme modulators.

Compared to other SIRT7 inhibitors identified from commercial libraries, our identified **YZL-51N** was an isolated natural compound from *P. americana* extracts. Different from other works, we particularly clarified the pharmacological mechanism of **YZL-51N**, which functions as a NAD^+^ competitive molecule to inhibit SIRT7. The NAD^+^-binding pocket of sirtuins is generally divided into three regions. Region A binds the adenine ribose moiety of NAD^+^, while region B binds nicotinamide ribose, and region C lies deep within the pocket.^26^^,^^49^^,^^50^ Interestingly, several SIRT7-**YZL-51N** interaction residues overlapped with SIRT7-NAD^+^ interaction residues in region A. Among them, D118A, N297A, L298A and C315A mutations demonstrated downregulated deacetylase activities as compared with the WT SIRT7. Even though SIRT1 and SIRT6 also locate in the nucleus like SIRT7, **YZL-51N** and NAD^+^ interact with SIRT1 or SIRT6 by different amino acids that may possibly lead to intermolecular force differences. In addition, we investigated different SIRT7 enzymatic activities, and found **YZL-51N** to be a SIRT7 inhibitor against diverse activities, which may be explained by the same coenzyme NAD^+^ that SIRT7 used for catalyzation. A similar cofactor competition strategy has also been used successfully to develop specific inhibitors for epigenetic modifiers. For example, compound **A-485** competed with acetyl-CoA to bind p300 and inhibit its acetylase activity, without affecting other histone acetylases such as PCAF, TIP60, and HAT1.^51^ In addition, compound EPZ005687, a potent EZH2 inhibitor, bound to the S-adenosylmethionine (SAM)-binding pocket of EZH2 and had 50-fold selectivity against EZH1.^52^

Post-translational modifications (PTMs) of histones play crucial roles in the structure and dynamics of chromatin, influencing various DNA-related biological processes.^53^ SIRT7 was found to participate in DNA repair through its deacetylation of multiple substrates in response to stimuli such as IR, ultraviolet (UV), or glucose starvation.^54^ Upon early IR exposure, SIRT7 is recruited to DSBs regions in a PARP1-dependent manner to facilitate H3K18ac deacetylation.^14^ In this study, we proved that **YZL-51N** treatment can suppress SIRT7-mediated H3K18ac deacetylation both *in vitro* and in different cancer cell lines. In addition, we previously demonstrated that SIRT7 directly interacts with and deacetylates ATM at Lys3016 residue during the late stage of DDR.^16^ Consistently, **YZL-51N** treatment led to persistent ATM acetylation and compromised DSB repair efficiency. It is possible that **YZL-51N** also inhibits SIRT7-mediated deacetylation of other targets, such as PCAF,^55^ GABPβ1,^56^ PAF53,^57^ NPM,^58^ SMAD4^59^ or ribosomal RNA,^60^ contributing to the anti-cancer effect of **YZL-51N**. Interestingly, SIRT7’s functionality extends beyond deacetylation to include desuccinylation,^15^ mono-ADP-ribosylation,^47^ debutyrylation,^45^ and deglutathionylation.^61^ Our findings revealed that compound **YZL-51N** treatment can suppress SIRT7-mediated H3K122succ desuccinylation, suggesting a possible role of **YZL-51N** in regulating chromatin relaxation after DNA damage. Similarly, **YZL-51N** seemed to cause a pan-butyrylation increase in histones, which may be associated with SIRT7-catalyzed histone debutyrylation.^45^ We consider that these effects of **YZL-51N** can at least in part be explained by the NAD^+^ competitive mechanism. In addition to DNA damage repair, there are other SIRT7-related cellular pathways that affect colon cancer cell proliferation and metastasis. For instance, SIRT7 upregulated CRC cells proliferation and invasion by repressing E-cadherin, and promoted hepatic and lung metastasis *in vivo* via ERK1/2 activation.^8^^,^^48^ The anti-cancer effect of **YZL-51N** is considered far more complex and related to various biological processes. Apart from our suggested combined chemo-radiotherapy, there may be other effective strategies dealing with cancer by using this compound. Considering the diverse physiological and pathological roles of SIRT7, **YZL-51N** and its derivatives are hopeful to be used for the treatment of other diseases in the future.

### Limitations of the study

Several attempts have been made to resolve the crystal structure of SIRT7 in order to gain a deeper understanding of its biological functions. To date, the N-terminal crystal structure of SIRT7 has been determined and revealed the presence of some helixes in a disordered region at 2.33 Å (Protein DataBank, PDB: 5IQZ).^62^ However, the SIRT7 crystal structure containing catalytic core domain and C-terminal domain has not been clarified. The C-terminal domain of SIRT7 was found essential for DNA-, RNA-, and nucleosome-regulated SIRT7 activity through positive-negative charge interactions.^63^^,^^64^^,^^65^ While addressing this challenge, we noted that although the C-terminal domain helped to expand SIRT7 soluble expression, its disordered region impeded crystallization. Further improvements will focus on N- and C-terminal sequence optimization to facilitate the formation of SIRT7 crystals with bound **YZL-51N**.

## STAR★Methods

### Key resources table

**Table:** 

REAGENT or RESOURCE	SOURCE	IDENTIFIER
**Antibodies**		

Rabbit Monoclonal anti-SIRT7	Cell Signaling Technology	Cat#sc-135055; RRID:AB_10611782
Rabbit polyclonal anti-SIRT6	Cell Signaling Technology	Cat#12486S; RRID:AB_2636969
Mouse Monoclonal anti-SIRT1	Santa Cruz Biotechnology	Cat#sc-74465; RRID:AB_1129462
Rabbit Polyclonal anti-H3K9ac	Abcam	Cat#ab4441; RRID:AB_2118292
Rabbit Monoclonal anti-H3K14ac	Abcam	Cat#ab52946; RRID:AB_880442
Rabbit Polyclonal anti-H3K18ac	Abcam	Cat#ab1191; RRID:AB_298692
Rabbit Polyclonal anti-Histone 3	Proteintech	Cat#17168-1-AP; RRID:AB_2716755
Rabbit Polyclonal anti-H3K122succ	PTM Bio	Cat#PTM-413; RRID:AB_2868522
Rabbit Monoclonal anti-ATM	Abcam	Cat#ab32420; RRID:AB_725574
Rabbit Monoclonal anti-ATM p1981	Abcam	Cat#ab81292; RRID:AB_1640207
Mouse Monoclonal anti-Tubulin	Santa Cruz Biotechnology	Cat#sc-398103; RRID:AB_2832217
Rabbit Polyclonal pan anti-Acetylation	Cell Signaling Technology	Cat#9441; RRID:AB_331805
Rabbit Polyclonal pan anti-Butyryllysine	PTM Bio	Cat#PTM-301; RRID:AB_2687946
Mouse Monoclonal pan anti-Crotonyllsine	PTM Bio	Cat#PTM-502; RRID:AB_2877695
Mouse Monoclonal pan anti-Sucinyllysine	PTM Bio	Cat#PTM-419; RRID:AB_2942099
Mouse Monoclonal anti-Poly ADP-ribosylation	Trevigen	Cat#4335-MC-100; RRID:AB_2572318
Mouse Monoclonal anti-His tag	Proteintech	Cat#HRP-66005; RRID:AB_2857904
Normal Mouse IgG secondary antibody	ZSGB-Bio	Cat#ZB-2305; RRID:AB_2747415
Normal Rabbit IgG secondary antibody	ZSGB-Bio	Cat#ZB-2301; RRID:AB_2747412

**Bacterial and virus strains**		

BL21(DE3) competent cells	Tiangen	Cat#CB106
DH5α competent cells	Tiangen	Cat#CB101

**Chemicals, peptides, and recombinant proteins**		

DMEM	Gibco	Cat#C11995500BT
Fetal Bovine Serum	ExCell Bio	Cat#FSP500
Trypsin	Macgene	Cat#K2708200
Penicillin/Streptomycin	Macgene	Cat#J3112200
4% Paraformaldehyde Fix Solution	DING GUO PROSPEROUS	Cat#AR-0211
NAD^+^	MCE	Cat#HY-B0445
NAM	Beyotime	Cat#S1761
Recombinant H3K18 acetylated peptides	AnaSpec	Cat#AS-64638-1
Triton X-100	Solarbio	Cat#T8200
PVDF membrane	Millipore	Cat#IPVH08100
SYPRO Orange	Thermo Fisher Scientific	Cat#S6651
UltraFection 3.0	4A Biotech	Cat#FXP135-100
Etoposide	Sigma-Aldrich	Cat#341205
Puromycin	InvivoGen	Cat#ANT-PR-1
Spindle poison colchicine	Sigma	Cat#3915
Giemsa stain	Baso Giemsa Stain	Cat#BA-4219
His-SIRT7	This manuscript	N/A
GST-SIRT1 (193-741)	Active Motif	Cat#7846-50
GST-SIRT2 (50-356)	Active Motif	Cat#31528
GST-SIRT3 (102-399)	Active Motif	Cat#31529
GST-SIRT4 (25-314)	Active Motif	Cat#31530
GST-SIRT5	Active Motif	Cat#31531
GST-SIRT6	Active Motif	Cat#31532

**Critical commercial assays**		

Sirtuin Activity Assay Kit	BioVision	Cat#K324
Pierce™ BCA Protein Assay Kits	Thermo Fisher Scientific	Cat#23225
CCK-8 reagent	Solarbio	Cat# CA1210
ECL kit	Millipore	Cat# WBKL50500
Sensor Chip CM5	Cytiva	Cat#BR100012

**Deposited data**		

MS, HPLC and NMR data et al.	This manuscript	Figures S1 and S15–S29
Original western blot images and microscope images	This manuscript	https://data.mendeley.com/datasets/rdzwdcbtyt/1

**Experimental models: Insect material**		

Dried whole *Periplaneta americana*	Gooddoctor Pharmaceutical Group	N/A

**Experimental models: Cell lines**		

HCT116	ATCC	Cat#CCL-247
HCT116 SIRT7-KO	Homemade	N/A
HT29	ATCC	Cat#HTB-38
SW620	ATCC	Cat#CCL-227

**Experimental models:Organisms/strains**		

BALB/c nude mice	Guangdong Yaokang Biotechnology Co. Ltd.	N/A

**Oligonucleotides**		

Primers sequence for mutant analysis	This manuscript	Table S1

**Recombinant DNA**		

His-SIRT7	This manuscript	N/A
pET-28a	Addgene	Cat# 69864-3
PGEX-4T-1	Addgene	Cat# 27458001
His-SIRT7-T112A	This manuscript	N/A
His-SIRT7-D118A	This manuscript	N/A
His-SIRT7-G268A	This manuscript	N/A
His-SIRT7-N297A	This manuscript	N/A
His-SIRT7-G298A	This manuscript	N/A
His-SIRT7-C315A	This manuscript	N/A

**Software and algorithms**		

GraphPad Prism 8	Graphpad	https://www.graphpad.com/scientific-software/prism/
Image J	NIH	https://imagej.nih.gov/ij/
BioRad ChemiDoc XRS+	BioRad	https://www.bio-rad.com/

### Resource availability

#### Lead contact

Further information and requests for resources and reagents should be directed to and will be fulfilled by the Lead Contact, Wei-Guo Zhu (zhuweiguo@szu.edu.cn).

#### Materials availability

This study did not generate new unique reagents. Plasmids and cell lines are listed in the key resources table and available for use upon request to the lead contact.

#### Data and code availability

Original western blot images and microscope images have been deposited at Mendeley Data. The Mendeley link is listed in the key resources table.

This paper does not report original code.

Any additional information required is available from the lead contact upon request.

### Experimental model and study participant details

#### Insect material

Dried whole *Periplaneta americana* were provided by Gooddoctor Pharmaceutical Group in Sichuan Province, PR China, in August 2015. A voucher specimen (CHYX-0594) was deposited at the School of Pharmaceutical Sciences, Shenzhen University Health Science Center, PR China.

#### Cell lines

HCT116, HT29 and SW620 cells were obtained from obtained from the American Type Culture Collection (ATCC, Manassas, Virginia, USA). SIRT7-KO cells used in this study were generated using the CRISPR-Cas9 gene targeting approach, in which the cells were infected with the gRNA-harbored lentivirus and single clones were selected with puromycin (2 mg/mL). All cells were maintained in high-glucose Dulbecco’s Modified Eagle’s Medium (DMEM) supplemented with 10% fetal bovine serum (FBS). The cells were incubated at 37°C in a humidified chamber containing 5% CO2. All cells used in this study were authenticated by short tandem repeat analysis and tested for mycoplasma contamination regularly.

#### Mouse models

The animal experiments described in this study were approved by Shenzhen University Animal Care and Use Committee and were conducted in accordance with guide for the care and use of laboratory animals. All male BALB/c nude mice (4 weeks, 14-16 g) purchased from Guangdong Yaokang Biotechnology Co. Ltd. The mice were housed under specific-pathogen-free conditions. The animal room had controlled temperature (18-23°C) and humidity (40-60%) and a 12 h light/12 h dark cycle.

### Method details

#### Plasmid construction and protein purification

His-tagged full length SIRT7 protein expression from BL21 bacterial cells was induced by the addition of 0.25 mM isopropyl β-D-1-thiogalactopyranoside (IPTG). The protein was then purified using a HisTrap™ HP 5-mL column in lysis buffer (25 mM Tris-HCl, 500 mM NaCl and 20 mM Imidazole pH 8.5), and then eluted in elution buffer (25 mM Tris-HCl, 500 mM NaCl and 500 mM Imidazole pH 8.5). The protein samples were passed through Superdex™ 75 (SD75) and resuspended in buffer (20 mM HEPES, 150 mM NaCl, pH 7.0) by AKTA Pure 25 M1 (Cytiva, USA). SIRT7 protein were snap-frozen in liquid nitrogen and stored at -80°C. GST-SIRT1-193-741, GST-SIRT2-50-356, GST-SIRT3-103-399, GST-SIRT4-25-314, full-length GST-SIRT5, and full-length GST-SIRT6 were purchased from Active Motif, Inc.

#### Fluor-de-Lys deacetylase assay

For *in vitro* analysis of SIRT7 deacetylation, the reaction system contained 10 μg His-SIRT7, 0.5 mM NAD^+^, 10 μM ARTKQTARKSTGGKAPRK(MCA)QLAGGK (synthesized by QYAOBIO; details in the supplementary files), 50 μM potential compound or 3 mM NAM, and assay buffer (10 mM Tris-HCl pH 8.0, 4 mM MgCl_2_, 0.2 mM DTT and 10% Glycerol). The deacetylation reactions were incubated at 37°C for 90 min. The fluorescence intensity was measured with a microplate reader (Synergy H4 Hybrid Reader, Biotek) at an excitation wavelength of 260 nm and detected at an emission spectrum of 300–600 nm. The IC_50_ values were calculated at 450 nm emission by GraphPad Prism 8.

#### Dot blot assay

For dot blot assays, the reaction system containing 10 μg His-SIRT7, 0.5 mM NAD^+^, 10 μM biotin-H3K18ac peptides and 50 μM potential compound was mixed and incubated at 37°C for 90 min. Samples were transferred to a polyvinylidene difluoride (PVDF) membrane, and then blocked with 5% skimmed milk. The blots were probed with H3K18ac primary antibody at RT for 2 h and then incubated with secondary antibody at RT for 1h. The bands were visualized using ECL substrates (Thermo Fisher, USA) on Tanon 5200SF Imaging System.

#### Sirtuin deacetylation assay

Deacetylase activity of 10 μg purified SIRT proteins was assessed using the Sirtuin Activity Assay Kit (BioVision, Cat#K324-100, USA) according to the manufacturer’s instructions. The fluorescence intensity (Ex/Em = 400/505 nm) was measured using a microplate reader (Synergy H4 Hybrid Reader, Biotek) in the end-point mode.

#### Biolayer interferometry assay

The binding affinity of SIRT1/2/6/7 with **YZL-51N** was determined using Super Streptavidin (SSA) biosensors in an Octet Red 96 instrument (ForteBio Inc., Menlo Park, CA, United States). Briefly, the biotinylated SIRT1/2/6/7 proteins were immobilized onto the surface of SSA biosensors. Increasing concentrations of **YZL-51N** were allowed to flow through the chips at room temperature in PBST buffer (pH 7.4). Assays were performed in black 96-well flat bottom plates on a shaker at 1000 r/min. The association and dissociation of **YZL-51N** with SIRT1/2/6/7 were measured for 300 s. Kinetic parameters and affinities were calculated from a non-linear global fit of the data between **YZL-51N** and SIRT1/2/6/7 using Octet Data Analysis software version 7.0 (Fortebio).

#### Protein thermal shift assay

The mixture containing SIRT1, SIRT6 or SIRT7 proteins, SYPRO Orange, compounds and buffer (100 mM Tris-HCl, 40 mM NaCl, 10 mM MgCl_2_, pH=7.4) were added into 0.2 mL PCR tubes for a total volume of 20 μL and placed in an ABI ViiA 7 Real-time PCR System. The temperature ranging from 35.0 to 75.0°C was increased at a rate of 0.5°C/s and the fluorescence intensity was monitored in the ROX channel.

#### Surface plasmon resonance

All SPR measurements were performed on Biacore T200 instrument (GE Healthcare) with running buffer (PBS+5% DMSO) at room temperature. The bacterially purified His-SIRT7 was covalently immobilized onto Series S Sensor Chip CM5 by a standard amino-coupling procedure in 10 mM sodium acetate (pH 5.5). Two-fold serial dilutions of **YZL-51N** or NAD^+^ in 5% DMSO passed through the sensor chip at 30 μL/min for 120 s, followed by 240 s of dissociation flow. The KD value was calculated based on the concentration dependence of the observed steady-state response using Biacore T200 Evaluation software Version 1.0 (GE Healthcare).

Using the same SIRT7 immobilized CM5 chip, SPR competitive binding assays were performed in the “A-B-A” model with the Biacore T200 instrument. NAD^+^ (0, 100, 500 and 2000 μM) were injected over the surface of the chip for 60 s at 30 μL/min. To test the competitive binding, 1 μM **YZL-51N** was mixed with the indicated concentrations of NAD^+^ to successively flow through SIRT7 immobilized chip. The responses were measured before the end of the injection.

#### Molecular modeling

The AlphaFold Protein Structure Database (https://alphafold.ebi.ac.uk/) was used to predict the potential structure of SIRT7 (Uniprot Q9NRC8). We then used grid-based ligand docking from energetics (GLIDE) software (Schrödinger suite 2009, v5.5) and PyMol program for virtual verification of the interaction of **YZL-51N** or NAD^+^ with the SIRT1/6/7 binding pocket and hot-spot amino acids.

#### Western blot analysis

After drug treatment, the cells were lysed in RIPA lysis buffer. Equal amounts of proteins were separated by sodium dodecyl sulfate polyacrylamide gel (7%–15%) electrophoresis (SDS-PAGE) and transferred to PVDF membranes. The membranes were then blocked with 5% skimmed milk in TBST and incubated overnight at 4°C with the primary antibodies followed by incubation with HRP-conjugated secondary antibodies. The protein bands were then detected using the ECL substrates and visualized with Tanon 5200SF Imaging System.

#### Cellular thermal shift assay

Cellular thermal shift assays were performed according to previously reported methods with some modifications.^66^ Briefly, HCT116 cells were lysed with RIPA buffer and incubated with 20 μM **YZL-51N** or DMSO for 1 h on ice. Subsequently, cell lysates were divided into 70 μL aliquots and heated individually at different temperatures for 4 min following by cooling on ice. The heated lysates were centrifuged, and 50 μL supernatants were taken for SDS-PAGE and immunoblotting analysis with the indicated antibodies.

#### HR and NHEJ assay

DSB repair efficiency was determined using cell-based reporter systems as described previously.^67^ DR-GFP or EJ5-GFP plasmids were stably transfected into HCT116 cells under the selection of puromycin. The cells were seeded in 6 well plates, infected with I-SceI virus for 18 h, re-cultured for 2 days and then treated with the indicated doses of **YZL-51N** for 12 h. GFP-positive cells were detected using a BD C6 Accuri flow cytometer (BD biosciences).

#### Comet assay

HCT116 cells were irradiated with 10 Gy IR and treated with 20 μM **YZL-51N**. Subsequently, 2 μL of a suspension of cells was mixed with 100 μL low melting point agarose and fixed on Comet slides for 30 min at 4°C. Then 200 mL lysis buffer (29.22 g NaCl, 7.44 g Na_2_EDTA, 0.24 g Tris, 1%DMSO, 1% Triton-X100, pH 10) was added to lysate the cells at 4°C for 2 h. The slides were lowered into comet assay running buffer (12 g/L NaOH, 0.37 g/L EDTA, pH 10) for 30 min before electrophoresis (20V, 200 mA, 30 min). The slides were stained with PI and washed twice with ddH_2_O. The images were viewed using Olympus IX73 fluorescence microscope.

#### Metaphase spreading

First, cells were seeded in 6 well plates for 24 h and then treated with 20 μM **YZL-51N** for 8 h or irradiated with 4 Gy IR. Subsequently, cells were treated with 0.4 μg/mL colchicine for 6 h. After the cells were harvested using trypsin, 2 mL 0.8% sodium citrate was added to make cells fragile. The cell samples were then resuspended in 5 mL Carnoy's fixative solution at RT for 15 min. The cells were centrifuged to remove excess fixative solution and dropped onto alcohol-cleaned slides. The slides were dried before incubated with Giemsa stain and then washed with ddH_2_O twice. Slides were scanned and photographed using the MetaSystem (Zeiss) in a light field using a 63× oil lens.

#### MTT assay

Cells were seeded into 96 well plates (4,000 cells/well). After 24 h, cells were treated with **YZL-51N** (0–160 μM) for 4 h before the addition of etoposide (0–80 μM) for 2 h at 37°C. The cells were then washed and re-cultured in fresh medium for 48 h. Cells were then treated with 10 μL 5 mg/mL MTT solution and incubated for 8 h at 37°C. Formazan were generated by the addition of 100 μL DMSO and shaking for 30 min. The absorbance of samples was measured at 570 nm using a microtiter plate reader (BioTek).

#### CCK-8 assay

Cells were seeded into 96 well plates (4,000 cells/well). After 24 h, cells were treated with **YZL-51N** (0–200 μM) for 48 h at 37°C. The plate was added with 10 μL CCK-8 solution to each well and incubated for 2 h at 37°C. The absorbance was measured at 450 nm using a microtiter plate reader (BioTek).

#### Colony formation assay

HCT116 cells were seeded into 60 mm dishes and treated with 0, 10 or 20 μM **YZL-51N** for 2 h and then exposed to 0, 2 and 4 Gy IR. After 4 h, the HCT116 cells (500 cells/well for 0 Gy, 1000 cells/well for 2 Gy, 2000 cells/well for 4 Gy) were seeded into 6 well plates and cultured for 14 days. Plates were washed twice with PBS before colonies were fixed in 4% formaldehyde and stained with 0.5% crystal violet for 1 h. Colonies were photographed using the ChemiDoc XRS^+^ Imager (Bio-Rad).

#### Animal experiment

For the xenograft models, HCT116 cells (5 x 10^6^ in 100 μL of PBS) were inoculated subcutaneously into the dorsal flank of the 4-week-old male BALB/c nude mice. The mice were randomly divided into control, drug treatment, IR treatment and combination treatment groups. Control group was injected with PBS. Drug treatment group was treated with 15 mg/kg **YZL-51N** every 3 d. In IR treatment group, the tumor site was irradiated with 3 Gy every 3 d. The combination group was treated with both 15 mg/kg **YZL-51N** and 3 Gy IR. The tumor size was estimated from the measurement of the longest diameter across the tumor and the corresponding perpendicular diameter. Tumor volumes were assessed on indicated days by a caliper and calculated using the formula: volume = (length x width^2^)/2.

### Quantification and statistical analysis

Data were presented as the mean ± SD as mentioned in the Figure legends. All experiments data were analyzed by using GraphPad Prism 8. After data distribution evaluation by Shapiro-Wilk test, for the parametric values > 0.05, unpaired two-tailed Student's t-tests were used for the comparison between two groups and ANOVA tests were used for multiple comparisons. For non-normal distribution, we opted for the kolmogorov-smirnov test. The statistical differences were considered significant at ∗p < 0.05; ∗∗p <0.01; ∗∗∗p < 0.001; ∗∗∗∗p < 0.0001; ns stands for no significant change. At least three independent replicates were performed for all experiments.
