# Supplementary material for: YZL-51N functions as a selective inhibitor of SIRT7 by NAD+ competition to impede DNA damage repair
Source: iScience. 2024 May 16;27(6):110014. doi: 10.1016/j.isci.2024.110014 (PMC11214487; doi:10.1016/j.isci.2024.110014)
Supplement: Document S1. Figures S1–S29 and Tables S1 and S2 [file mmc1.pdf]

iScience, Volume *27*

Supplemental information

YZL-51N functions as a selective inhibitor of SIRT7 by NAD^+ competition to impede DNA damage repair

Tian-Shu Kang, Yong-Ming Yan, Yuan Tian, Jun Zhang, Minghui Zhang, Yuxin Shu, Jinbo Huang, Jing He, Cheng-Tian Tao, Qian Zhu, Jinke Gu, Xiaopeng Lu, Yong-Xian Cheng, and Wei-Guo Zhu

Supplemental Figures and Legends

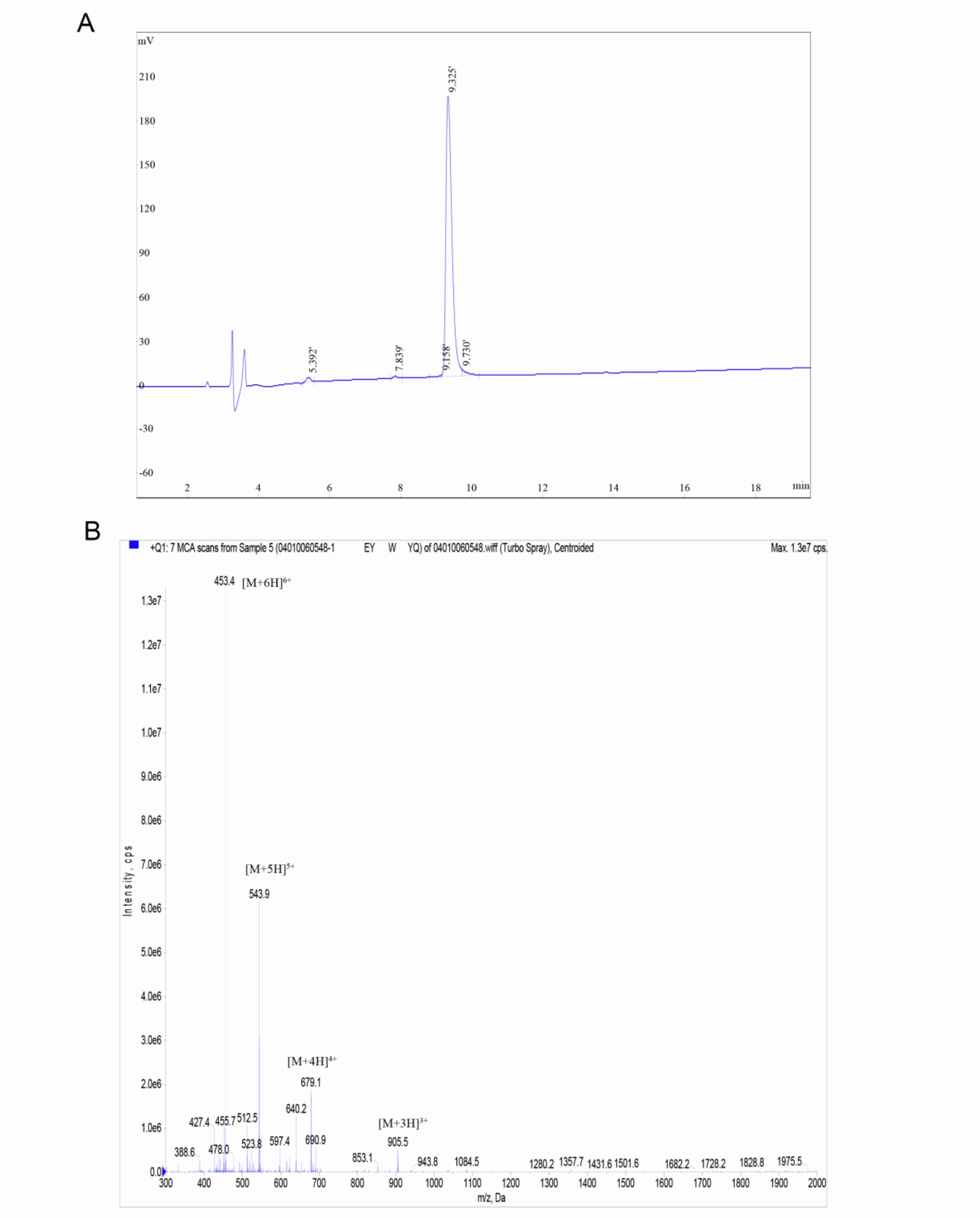

Figure S1. Confirmation of the synthesized peptides, related to Figure 1.

(A) HPLC chromatograms of the synthesized peptides with a UV detection at wavelength of 220 nm.
(B) The corresponding MS traces of the synthesized peptides.

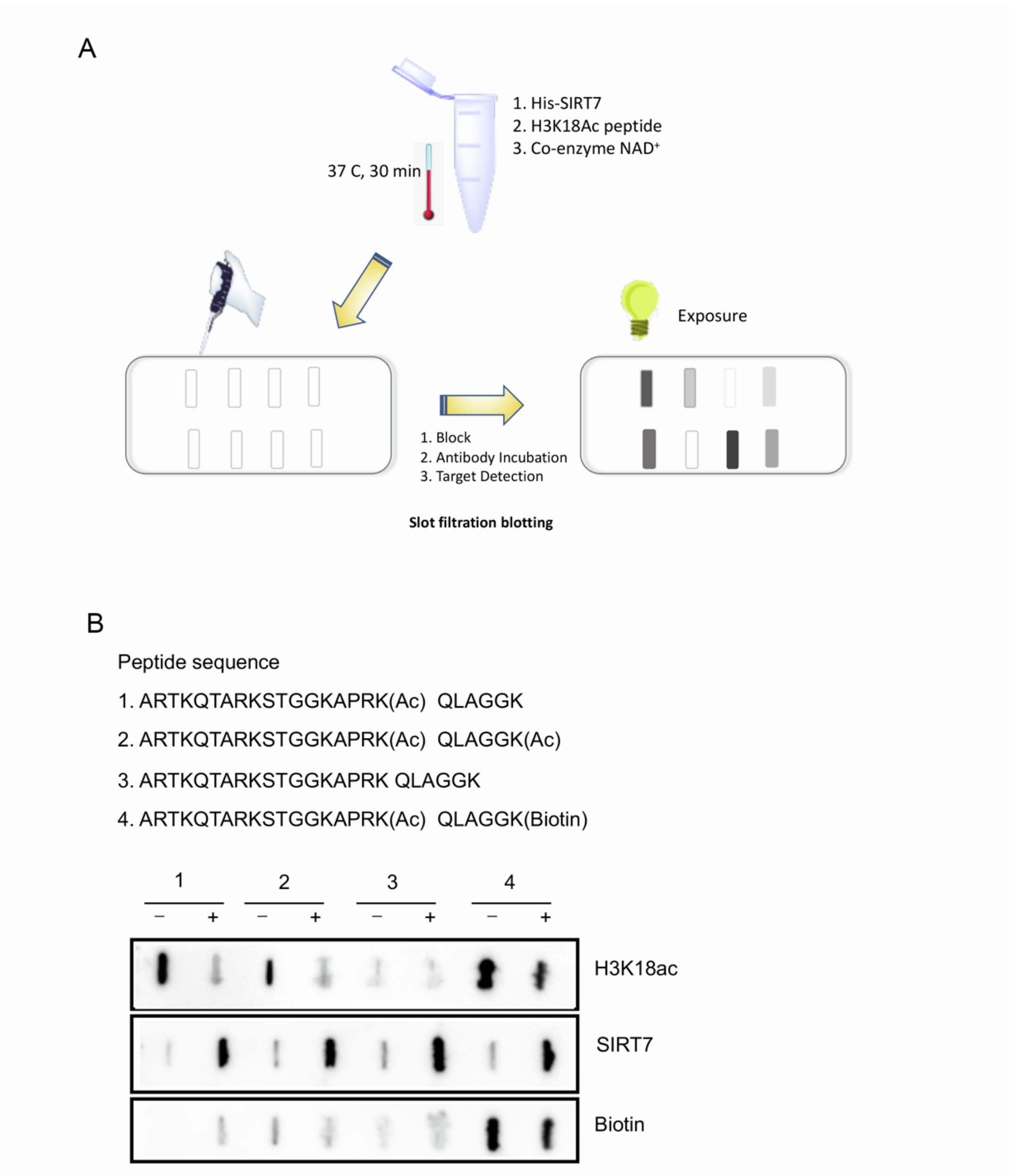

Figure S2. SIRT7 deacetylates histone H3 peptides in vitro, related to Figure 1.

(A) Schematic diagram of a dot blot assay. A mixture of purified His-SIRT7, peptide and NAD^+ were incubated at 37°C for 90 min and transferred to polyvinylidene difluoride (PVDF) membranes. The membranes were then blocked with 5% milk and detected with specific antibodies. (B) Dot blot assays were performed to detect the levels of H3K18ac, SIRT7 and Biotin.

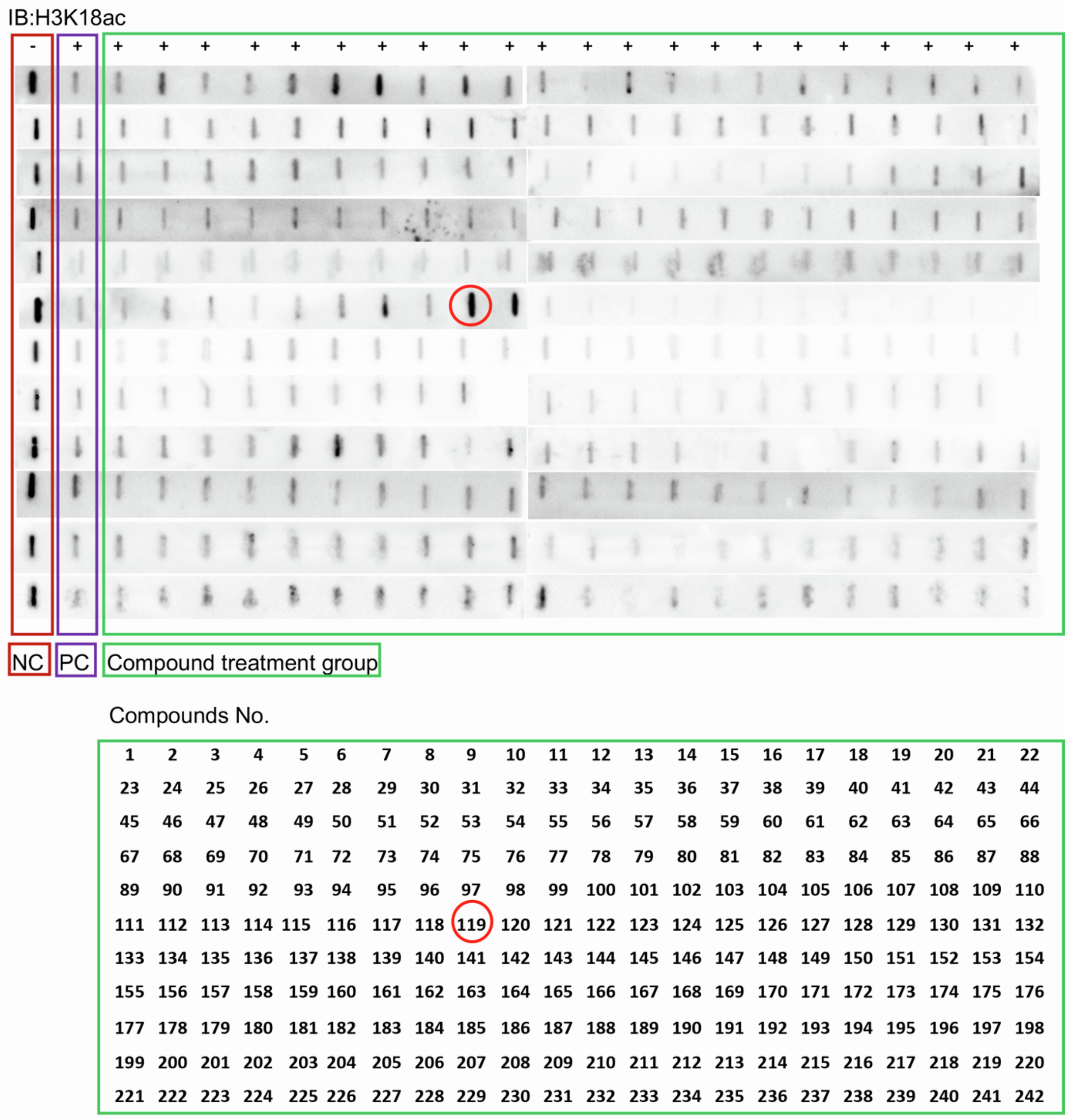

Figure S3. SIRT7 inhibitors screening by dot blot assays, related to Figure 1.

Dot blot assays were conducted to detect H3K18ac changes by 242 compounds. The reactions were incubated with 50 μM compounds for 90 min at 37°C. The red frames indicate the NC group with H3K18ac peptide alone; The purple frames indicate the PC group with H3K18ac peptide, SIRT7, and 0.1% DMSO; The green frames indicate the compound treatment group with H3K18ac peptide, SIRT7, and 50 μM compound. Red circle indicates the compound **YZL-51N** (compound No. **119**).

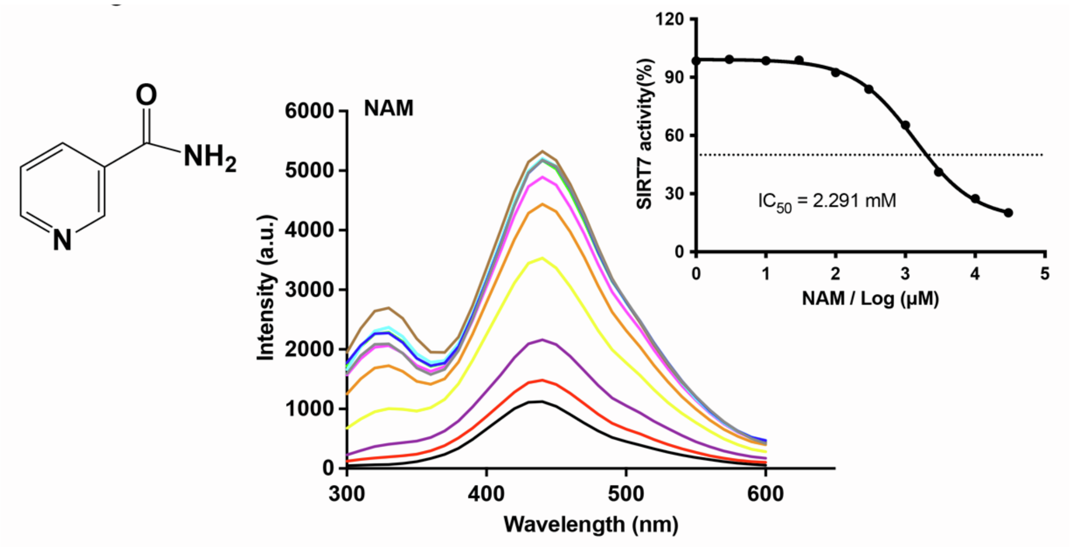

Figure S4. Chemical structure of NAM and inhibitory effects on SIRT7 deacetylase activity, related to Figure 1.

A mixture of purified His-SIRT7, H3K18ac peptide, NAD^+ and serial concentrations of (0-30 mM) NAM were incubated at 37°C for 90 min. Insert: Dose-dependent inhibitory effect of NAM on SIRT7 activity as measured by FDL assay. IC_{50} value is approximately 2.291 mM in this system.

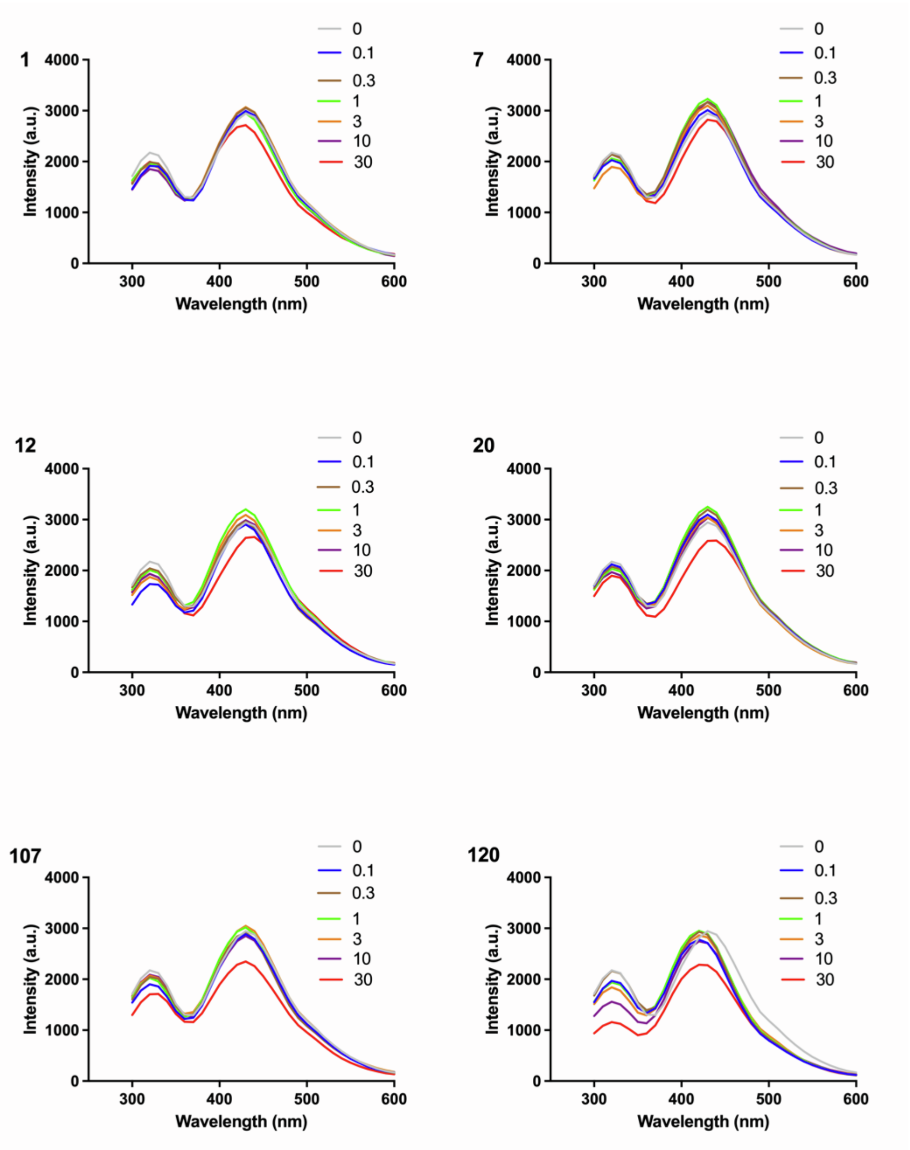

Figure S5. The inhibitory effects of compounds on SIRT7 deacetylase activity measured by FDL assays, related to Figure 1.

Fluorescence spectra revealed compounds-mediated fluorescent signals reduction in the presence of SIRT7, peptide substrates and NAD^+.

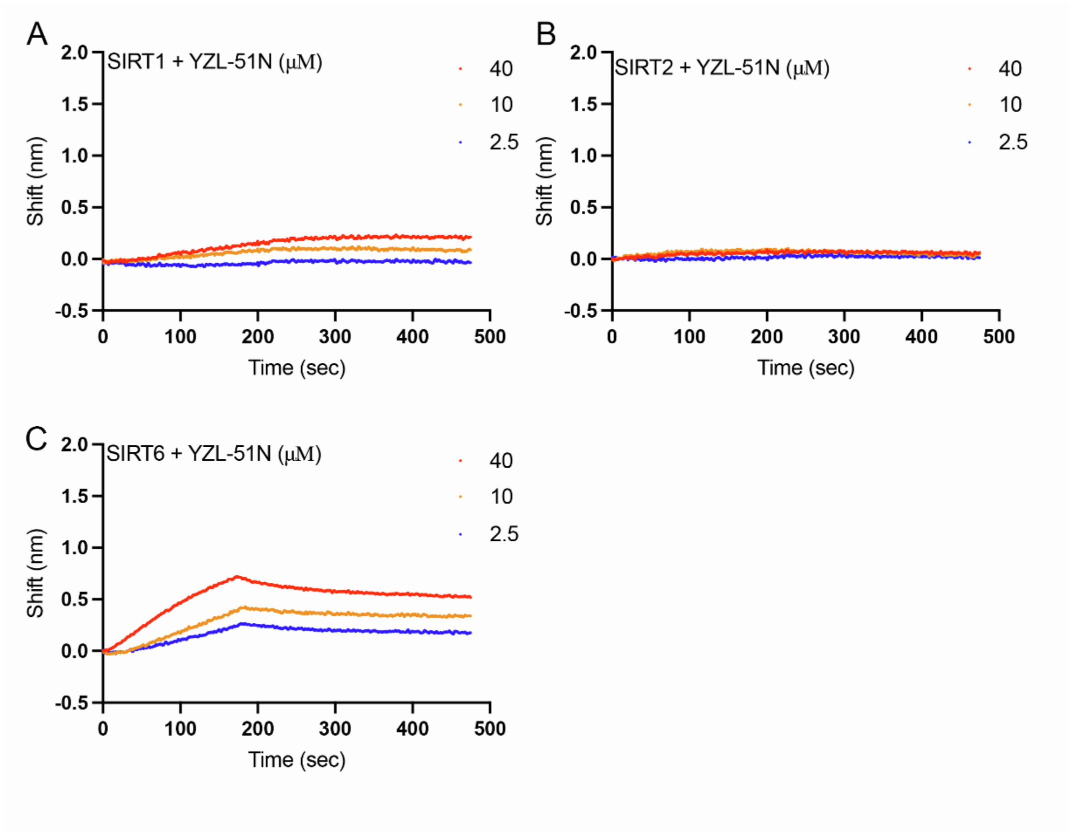

Figure S6. The binding affinity of YZL-51N to SIRT1, SIRT2 or SIRT6 measured by BLI assay, related to Figure 2.

The biotinylated SIRT1/2/6 proteins were immobilized onto the surface of SSA biosensors. 0-40 μM **YZL-51N** were allowed to flow through the chip at room temperature in PBST buffer (pH 7.4). Kinetic parameters and affinities were calculated using Octet Data Analysis software version 7.0 (Fortebio).

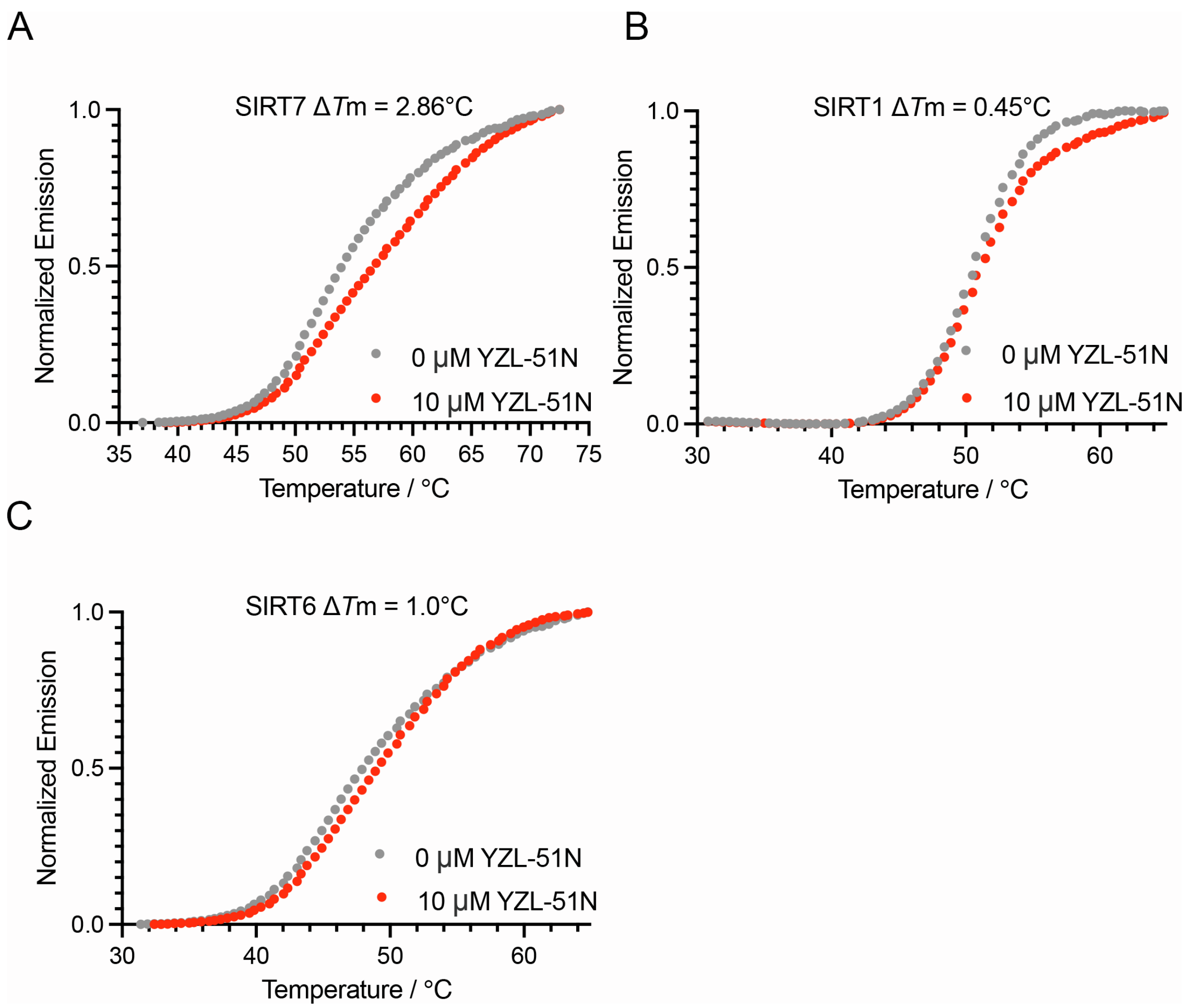

Figure S7. Thermostability analysis of SIRT1, SIRT6 and SIRT7 with YZL-51N, related to Figure 2.

Protein thermal shift assays were conducted to show the changes in thermostability of SIRT1, SIRT6 and SIRT7 with **YZL-51N**. The fluorescence intensity was monitored in the ROX channel.

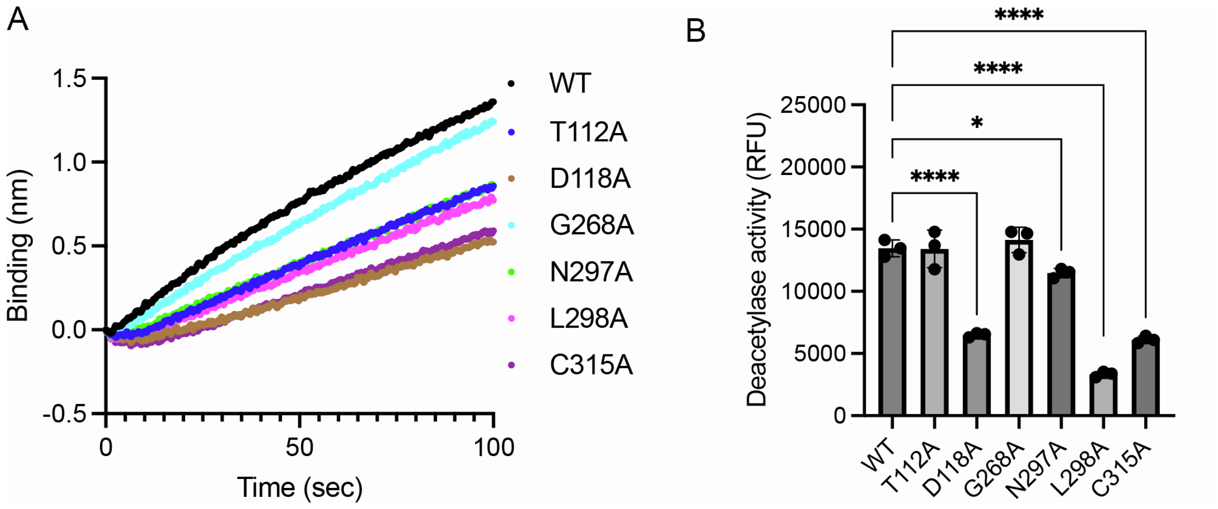

Figure S8. SIRT7 mutations decreased the binding affinity with YZL-51N and impaired its deacetylase activity, related to Figure 4.

(A) Biotinylated SIRT7 WT or mutant proteins were immobilized onto the surface of SSA biosensors. 40 μM **YZL-51N** were allowed to flow through the chips at room temperature in PBST buffer (pH 7.4). The binding signals were collected by using Octet Data Analysis software version 7.0 (Fortebio). (B) Fluorometric ELISAs were performed to compare the deacetylase activities of 10 μg recombinant SIRT7 WT with the mutant proteins. Data were presented as the mean ± SD of three independent experiments. Statistical analyses were performed using one-way ANOVA test. *$p < 0.05$; ****$p < 0.0001$.

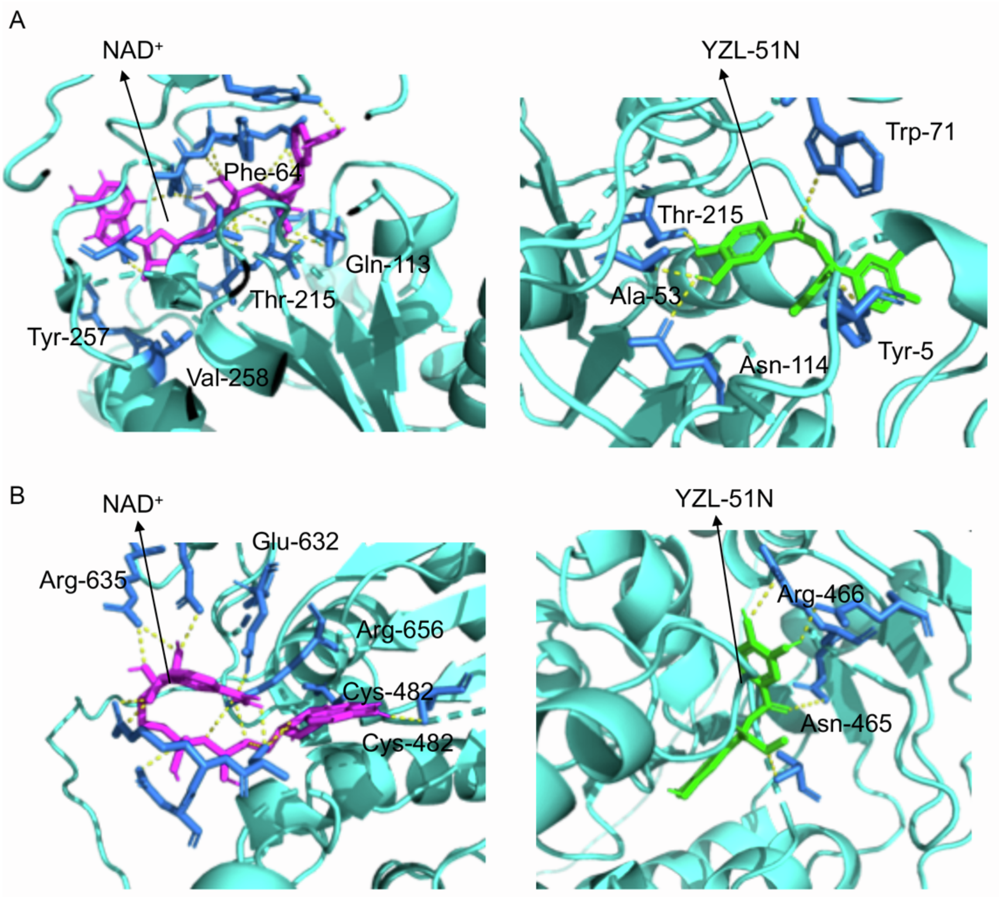

Figure. S9 Molecular docking of YZL-51N and NAD^+ to SIRT1 and SIRT6 by using AutoDock 4.2, related to Figure 4.

(A) Three dimensional structures of ligand-protein prediction showed NAD^+ (Left in panel) or **YZL-51N** (Right in panel) bound to SIRT1 and interacted with potential amino acids. (B) NAD^+ (Left in panel) or **YZL-51N** (Right in panel) bound to SIRT6 and interacted with potential amino acids.

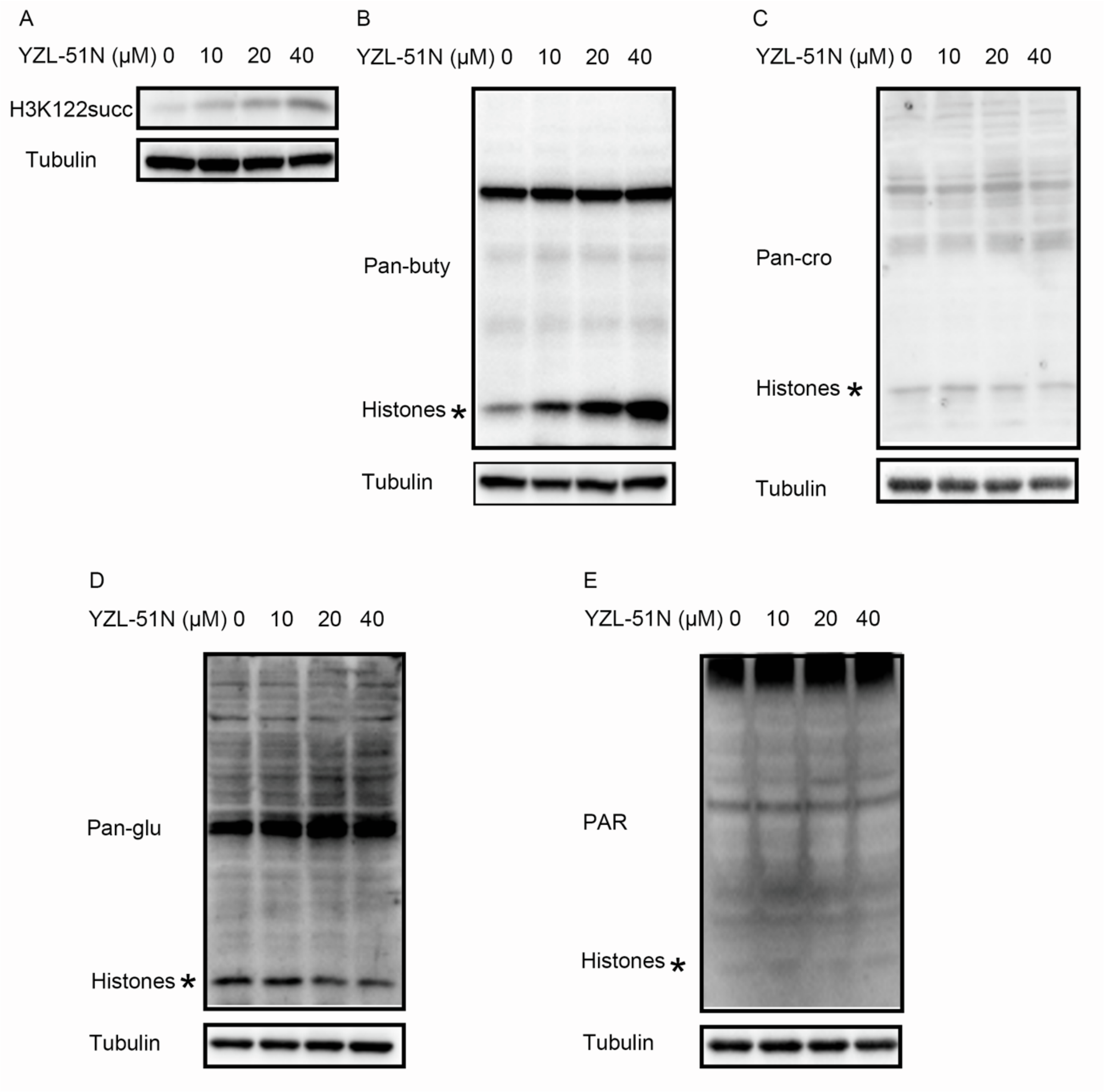

Figure S10. YZL-51N inhibits multiple enzymatic activities of SIRT7, related to Figure 5.

HCT116 cells were treated with 0-40 μM **YZL-51N** for 8 h, and the whole cell lysates were extracted to detect the levels of H3K122succ (A), pan-butyrylation (B), pan-crotonylation (C), pan-glutarylation (D) and PARylation (E). * indicates the position of different histones.

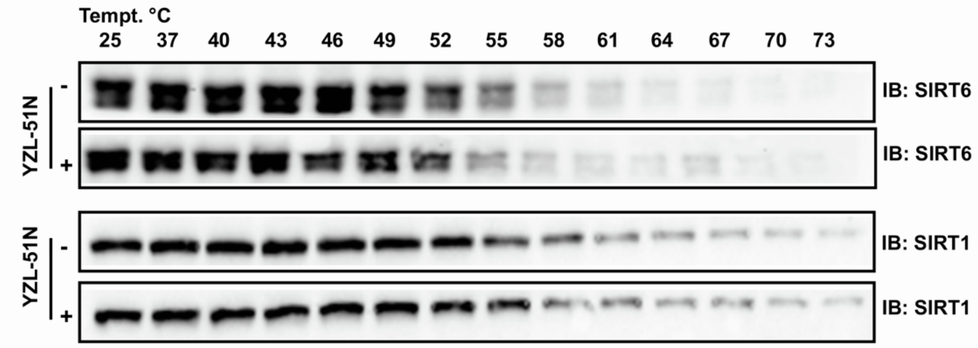

Figure S11. Cellular thermal shift assay used to detect the thermostability of SIRT1 and SIRT6 incubated with or without 20 μM YZL-51N, related to Figure 5.

HCT116 cell lysates were incubated with/without 20 μM **YZl-51N** at a temperature range. The thermostability changes of SIRT1 and SIRT6 were detected with western blot.

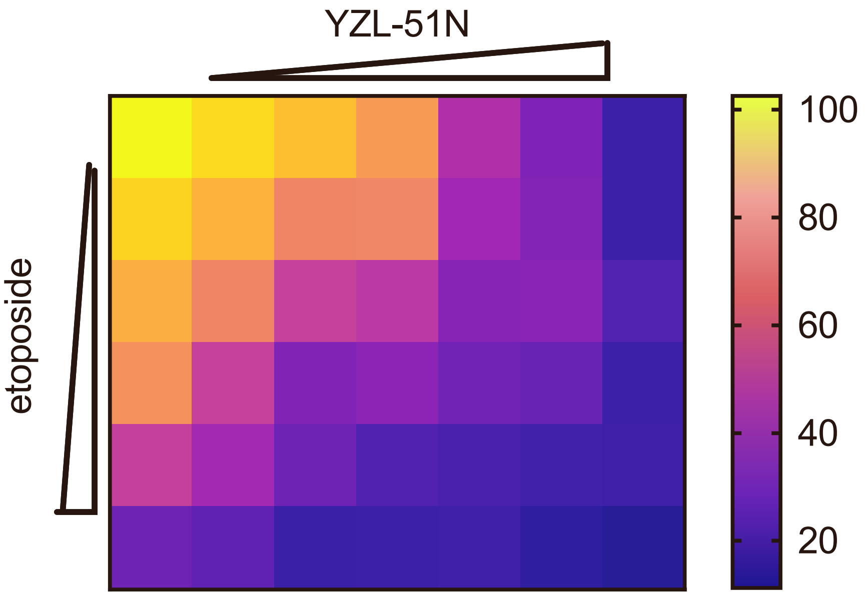

Figure S12. YZL-51N and etoposide combination chemotherapy against colorectal cancer cells, related to Figure 7.

Heat map of (0–160 μM) **YZL-51N** and (0–80 μM) etoposide combination therapy against HCT116 cells proliferation by MTT assay. The combination index for **YZL-51N** and etoposide can be calculated as following: $CI = \frac{(D)1}{(Dx)1} + \frac{(D)2}{(Dx)2}$, where $(Dx)_1$ represents the dose of **YZL-51N** alone that inhibits the growth of cells by x% and $(Dx)_2$ is the dose of etoposide alone that inhibits the growth of cells by x% (as shown in Table 1).

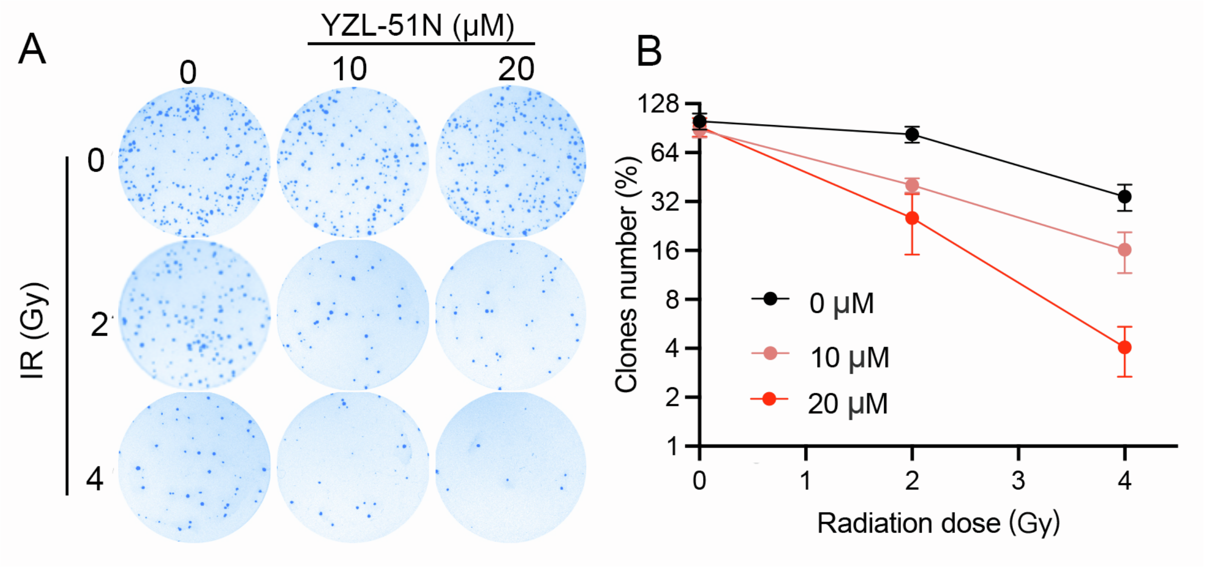

Figure S13. YZL-51N and IR combination against colorectal cancer cells, related to Figure 7.

(A) HCT116 cells were treated with 0-20 μM **YZL-51N** and irradiated with 0-4 Gy IR. For control group, 500 cells were seeded in each well. For IR treatment group,1000 or 2000 cells were seeded in each well. Cell clones were counted after 14 d cultivation. Statistical analyses were shown in (B). Data represent the mean ± s.d. of three independent experiments.

Property	Model Name	Predicted Value	Unit
Absorption	Water solubility	-3.334	log mol/L
Absorption	Caco2 permeability	0.42	log Papp in 10-6 cm/s
Absorption	Intestinal absorption	77.099	% Absorbed
Absorption	Skin Permeability	-2.735	log Kp
Absorption	P-glycoprotein substrate	Yes	
Absorption	P-glycoprotein I inhibitor	No	
Absorption	P-glycoprotein II inhibitor	No	

Property	Model Name	Predicted Value	Unit
Metabolism	CYP2D6 substrate	No	
Metabolism	CYP3A4 substrate	No	
Metabolism	CYP1A2 inhibitor	No	
Metabolism	CYP2C19 inhibitor	No	
Metabolism	CYP2C9 inhibitor	No	
Metabolism	CYP2D6 inhibitor	No	
Metabolism	CYP3A4 inhibitor	No	

Property	Model Name	Predicted Value	Unit
Excretion	Total Clearance	0.164	log ml/min/kg
Excretion	Renal OCT2 substrate	No	

Property	Model Name	Predicted Value	Unit
DIstribution	VDss (human)	0.182	log L/kg
DIstribution	Fraction unbound	0.16	Fu
DIstribution	BBB permeability	-1.27	log BB
DIstribution	CNS permeability	-3.619	log PS

Figure S14. The bioinformatical ADME evaluation of YZL-51N via pkCSM platform, related to Figure 7.

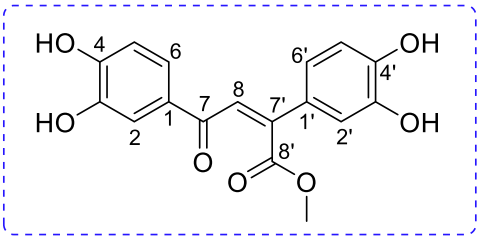

Figure S15. The structure of YZL-51N, related to Figure 3.

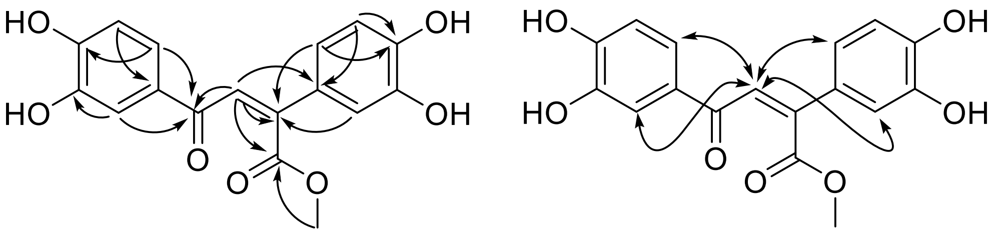

Figure S16. The key HMBC (↷) and ROESY (↔) correlations of YZL-51N, related to Figure 3.

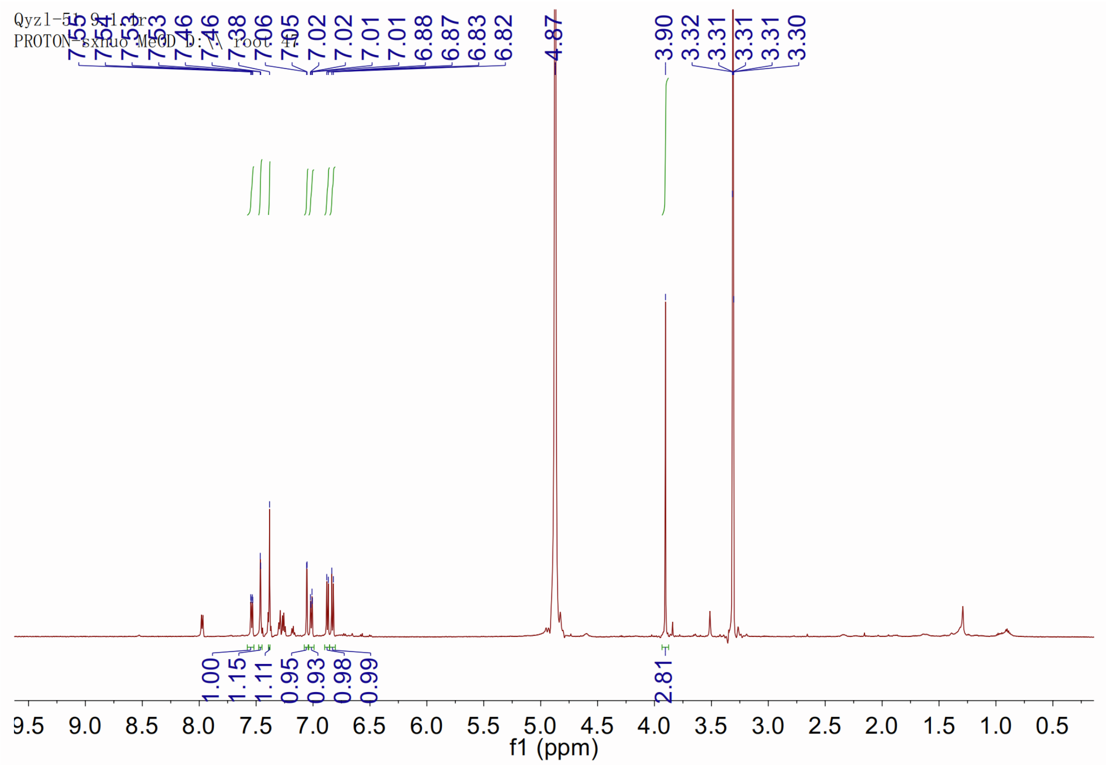

Figure S17. ^{1}H NMR spectrum of YZL-51N in CD_3OD, related to Figure 3.

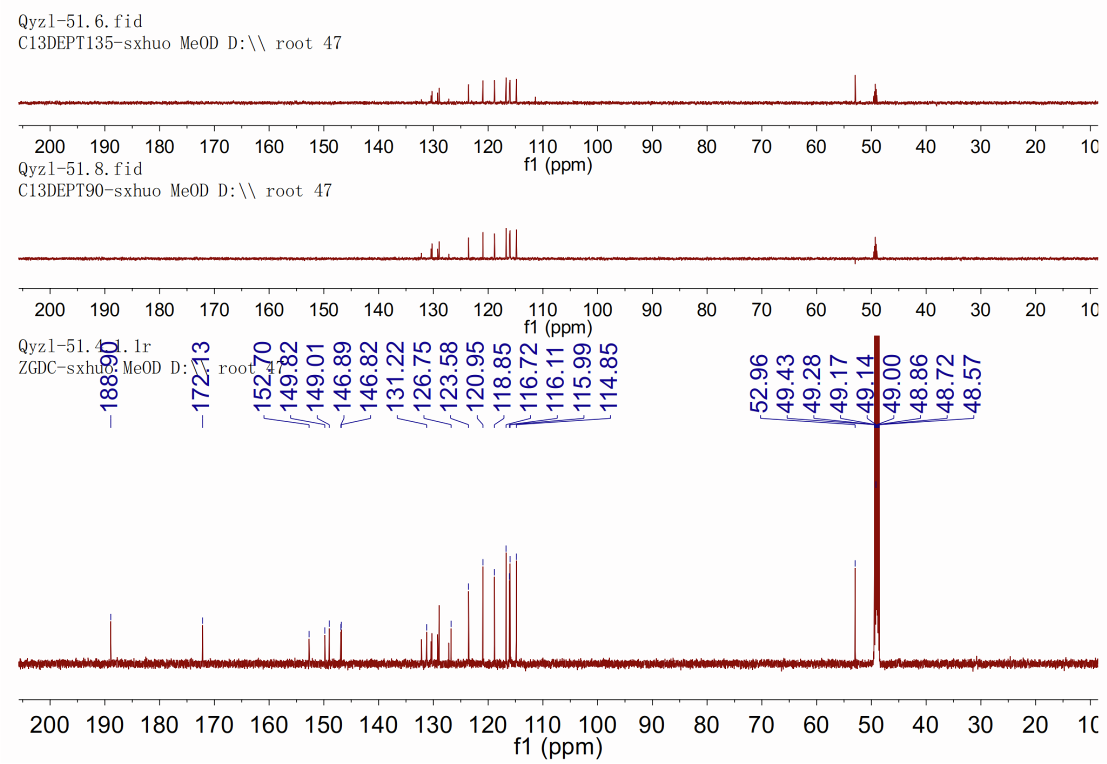

Figure S18. ^{13}C NMR and DEPT spectra of YZL-51N in CD_3OD, related to Figure 3.

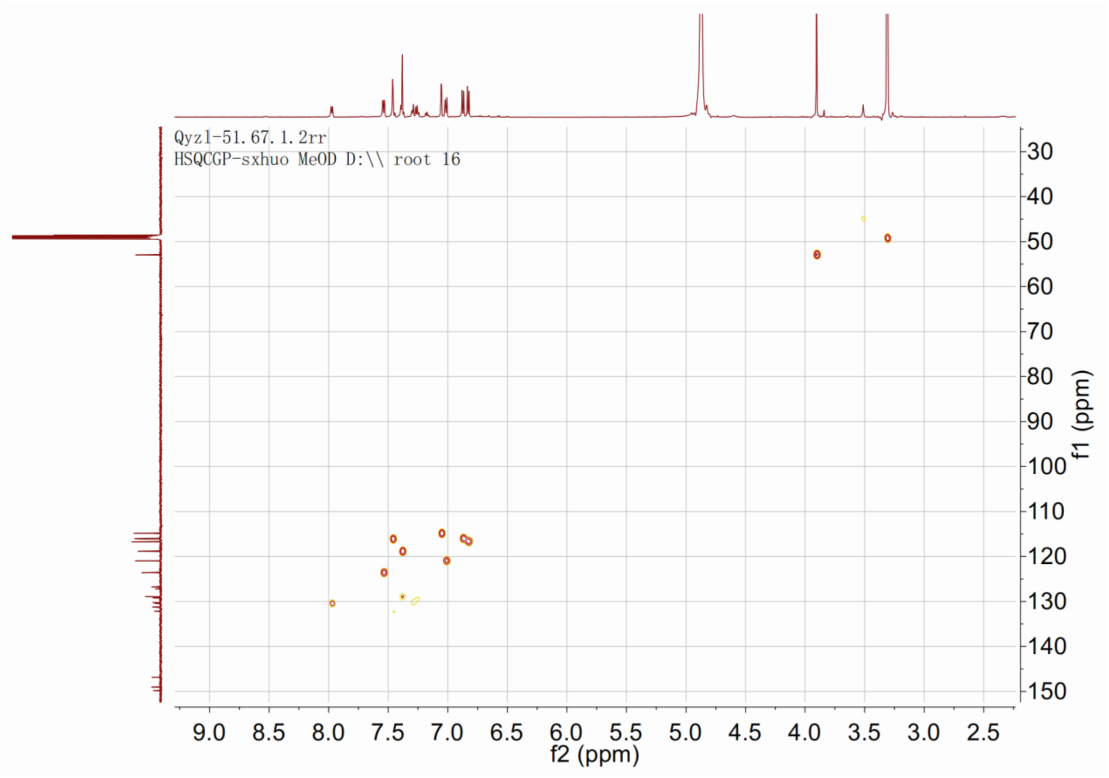

Figure S19. HSQC spectrum of YZL-51N in CD_3OD, related to Figure 3.

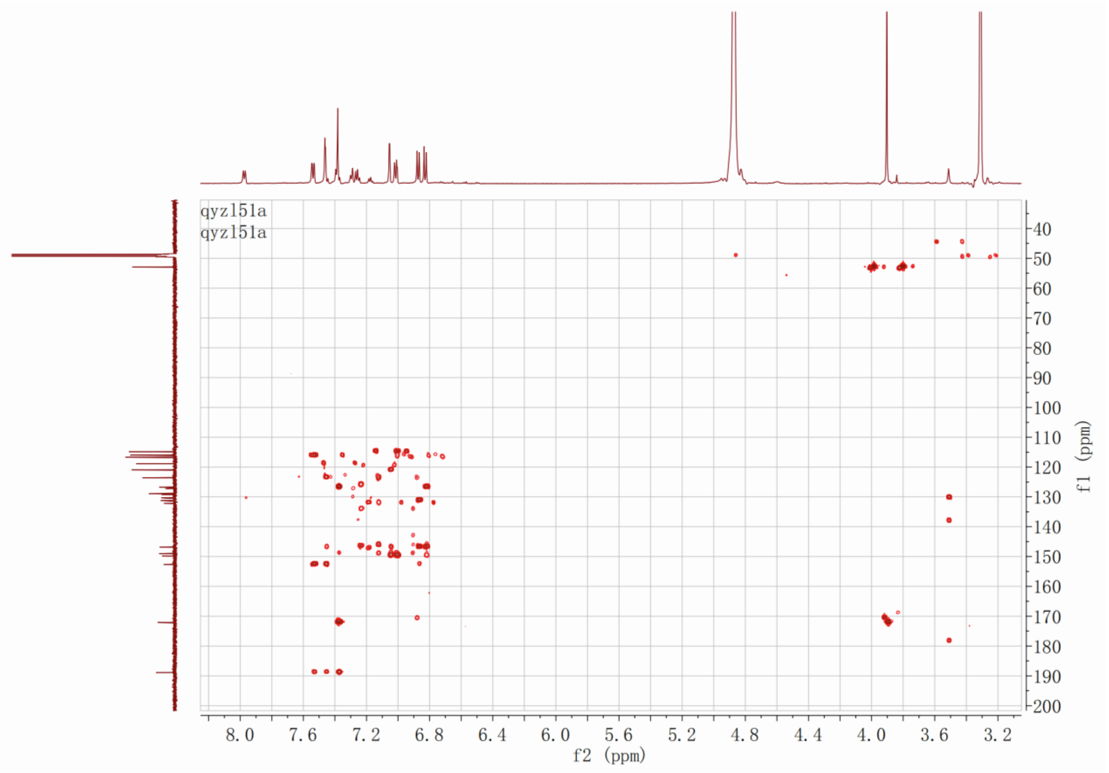

Figure S20. HMBC spectrum of YZL-51N in CD_3OD, related to Figure 3.

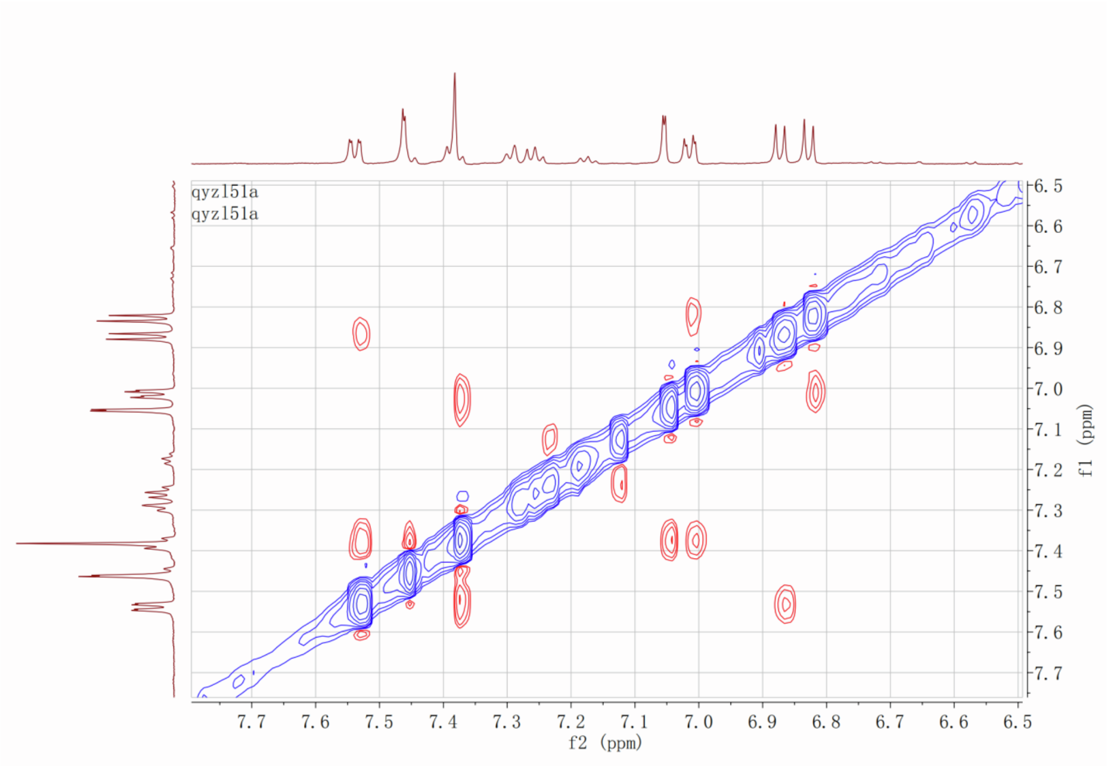

Figure S21. Key ROESY spectrum of YZL-51N in CD_3OD, related to Figure 3.

Formula Predictor Report - Qyzl-51a.lcd

Data File: E:\DATA\2017\0825\Qyzl-51a.lcd

Elmt	Val.	Min	Max	Elmt	Val.	Min	Max	Elmt	Val.	Min	Max	Elmt	Val.	Min	Max	Use Adduct
H	1	0	150	O	2	0	50	P	3	0	0	Br	1	0	0	H
B	3	0	0	F	1	0	0	S	2	0	0	I	3	0	0	
C	4	0	50	Na	1	0	0	Cl	1	0	0	Pt	2	0	0	
N	3	0	20	Mg	2	0	0	Fe	2	0	0					

Error Margin (ppm): 10
HC Ratio: unlimited
Max Isotopes: all
MSn Iso RI (%): 75.00
DBE Range: -2.0 - 100.0
Apply N Rule: yes
Isotope RI (%): 1.00
MSn Logic Mode: AND
Electron Ions: both
Use MSn Info: yes
Isotope Res: 10000
Max Results: 10

Event#: 2 MS(E-) Ret. Time : 0.550 Scan# : 112

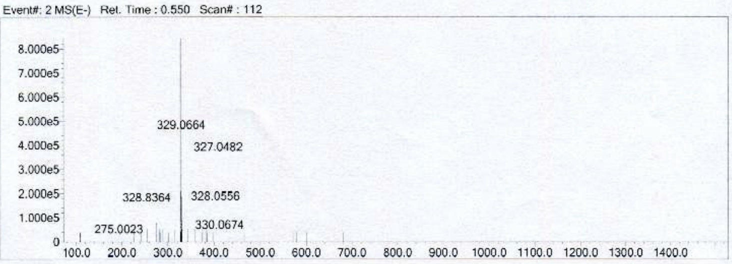

Measured region for 329.0664 m/z

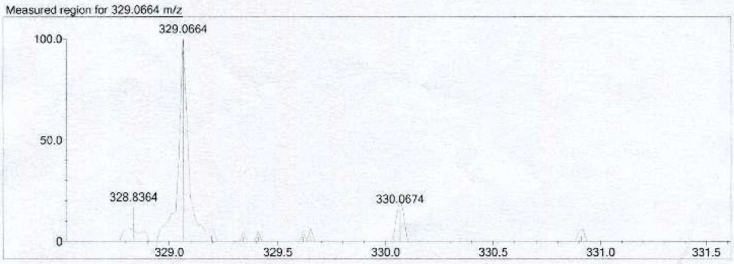

C17 H14 O7 [M-H]- : Predicted region for 329.0667 m/z

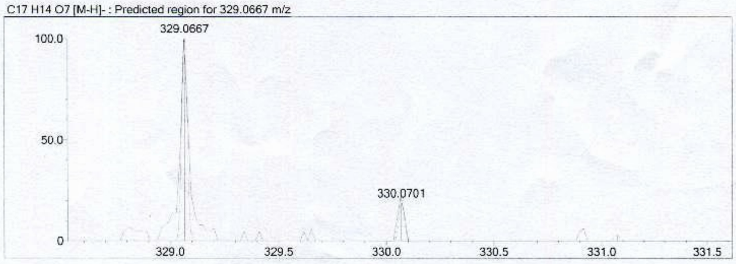

Formula (M)	Ion	Meas. m/z	Pred. m/z	Df. (mDa)	Df. (ppm)	DBE
C17 H14 O7	[M-H]-	329.0664	329.0667	-0.3	-0.91	11.0

Figure S22. HRESIMS of YZL-51N, related to Figure 3.

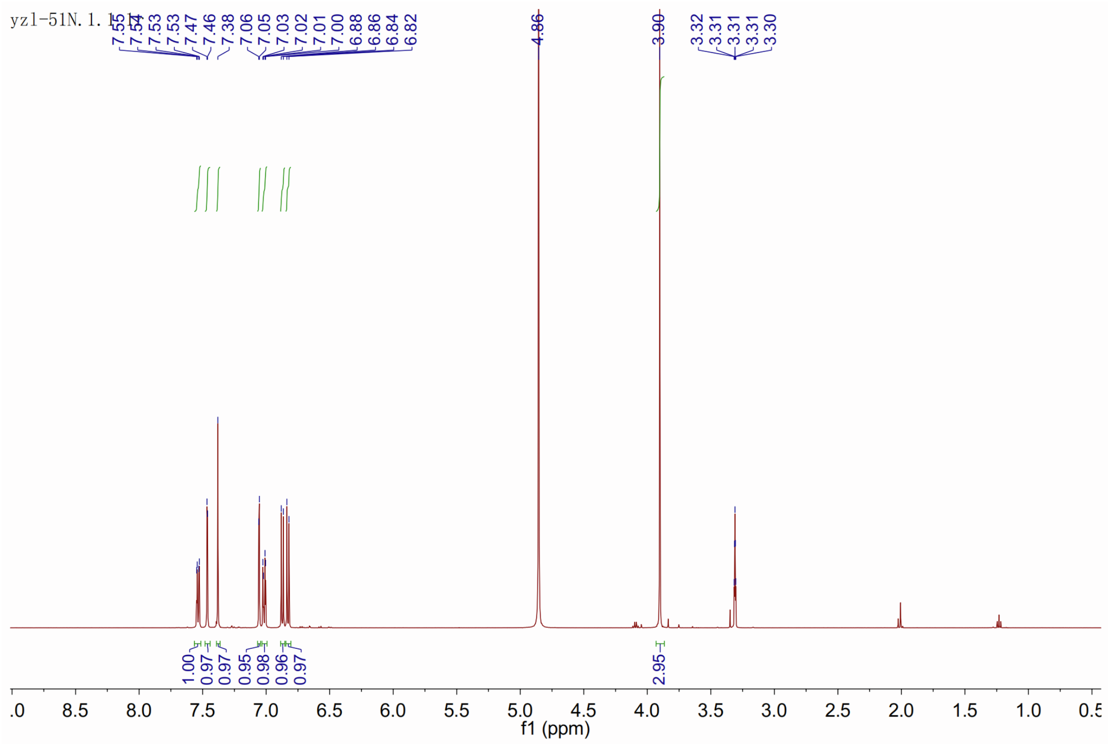

Figure S23. ^{1}H NMR spectrum of synthetic YZL-51N in CD_3OD, related to Figure 3.

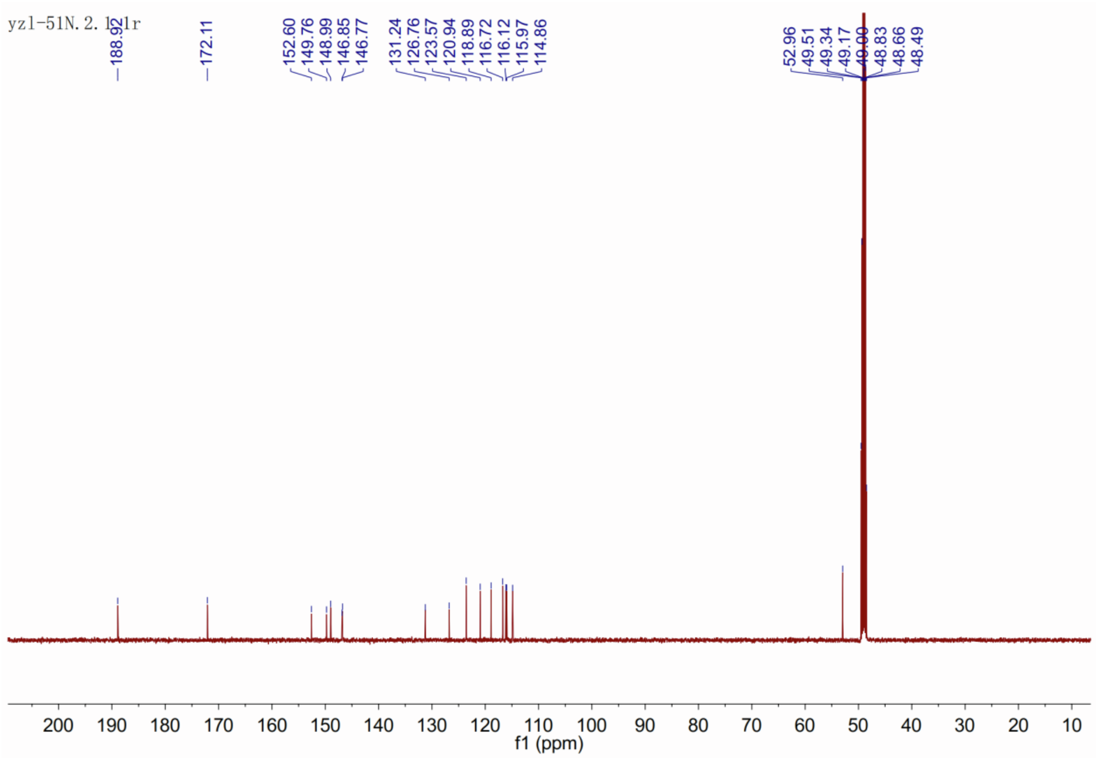

Figure S24. ^{13}C NMR spectrum of synthetic YZL-51N in CD_3OD, related to Figure 3.

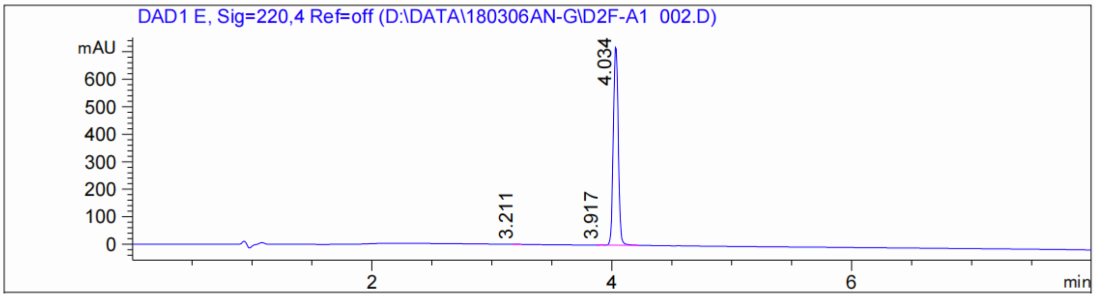

Figure S25. Synthetic YZL-51N was analyzed by HPLC (column: Phenomenex luna C18 250*10mm*10 μm; mobile phase: [water (0.1%TFA)-ACN]; B%: 3%-33%, 10 min), related to Figure 3.

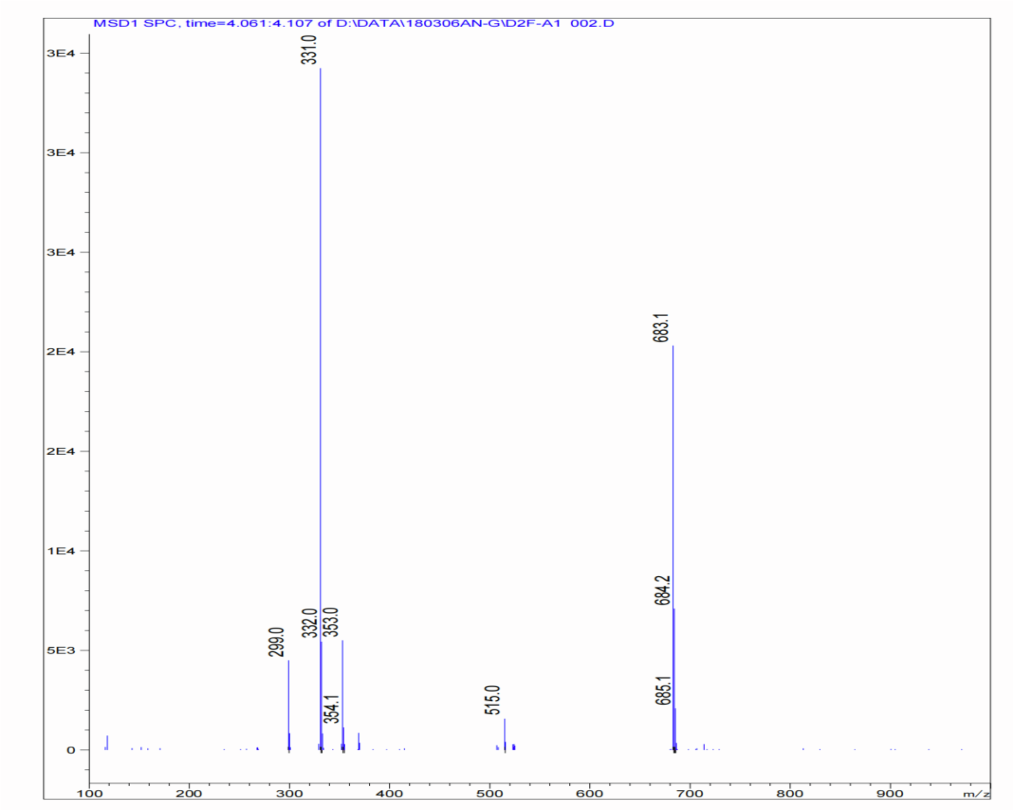

Figure S26. ESIMS of synthetic YZL-51N, related to Figure 3.

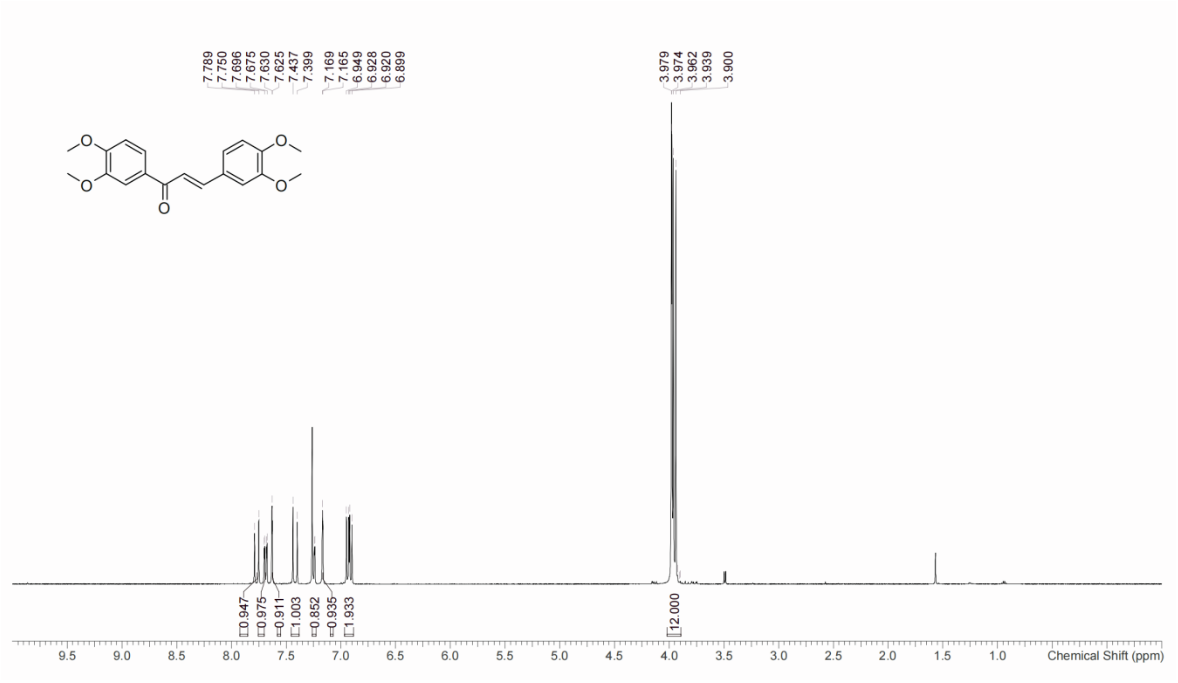

Figure S27. ^{1}H NMR spectrum of synthetic compound 2 in $CDCl_3$, related to Figure 3.

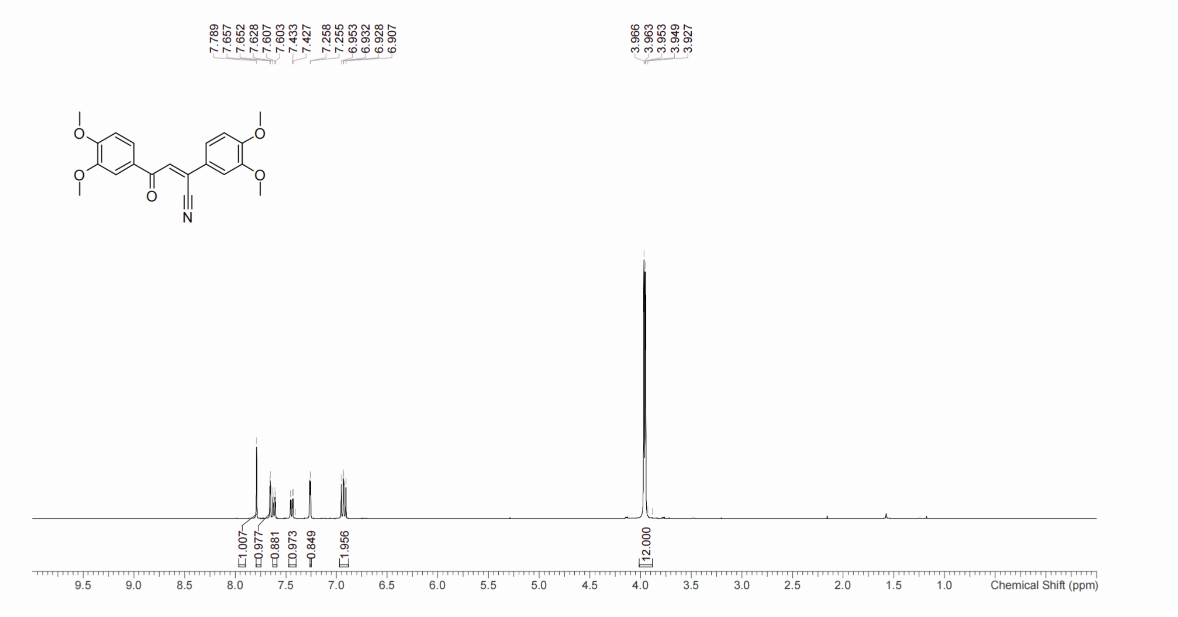

Figure S28. ^{1}H NMR spectrum of synthetic compound 4 in $CDCl_3$, related to Figure 3.

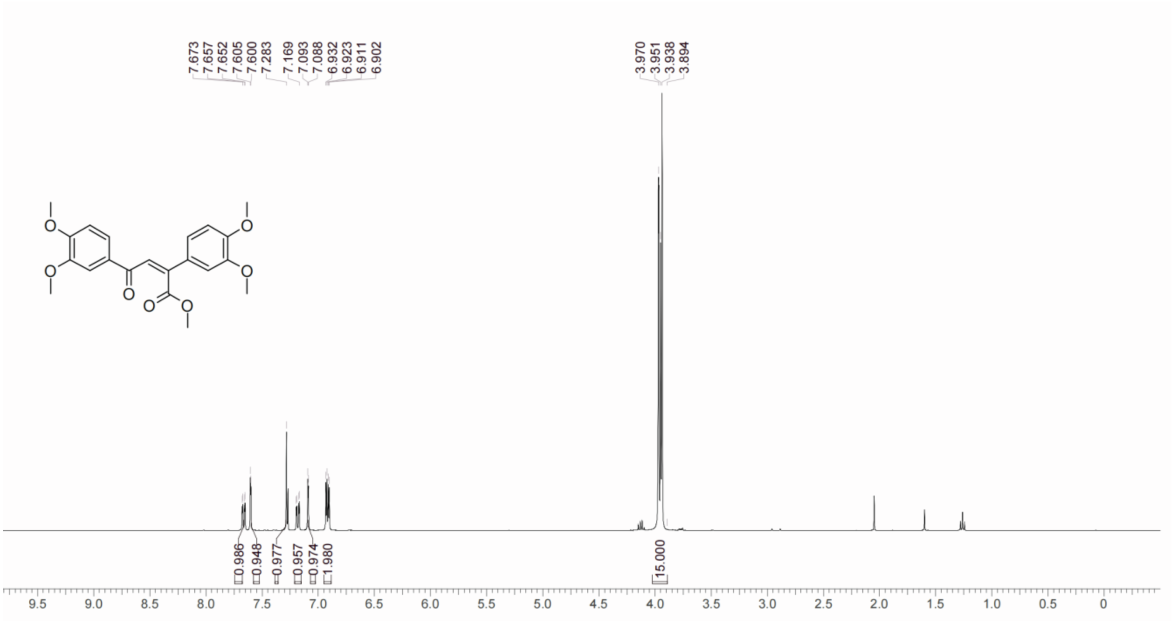

Figure S29. ^{1}**H NMR spectrum of synthetic compound 6 in** $CDCl_3$**, related to Figure 3.**

Table S1. Primers for molecular cloning on mutant analysis, related Figure 4.

Primers	sequences（5’ to 3’）
298A-F-61	ATCGTGAACGCTCAGTGGACC
298A-R-59	CCACTGAGCGTTCACGATGTA
297A-F-63	ATCGTGGCTCTGCAGTGGACC
297A-R-61	CCACTGCAGAGCCACGATGTA
Sirt7-F-61	CCTGTTTGGTCACTGATGCCT
Sirt7-R-54	CATCAGTGACCAAACAGGAAA
268A-F-59	CTGTGTCTAGCATCCAGCCTG
268A-R-58	GCTGGATGCTAGACACAGGAT
315A-F-59	CATGGGAAGGCTGATGACGTC
315A-R-57	GTCATCAGCCTTCCCATGTAG
112A-F-61	GCGGGAATCAGCGCTGCA
112A-R-68	TGCAGCGCTGATTCCCGCGC
118A-F-66	ATCCCAGCTTACCGGGGCCCT
118A-R-60	GCCCCGGTAAGCTGGGATAGA

Table S2. YZL-51N and etoposide combination index values against HCT116 cells, related to Figure 7.

(%, Mean ± SD, * means CI < 1, synergism; Δ, CI=1, Additive effect; CI > 1, Antagonism)

	0	5	10	20	40	80	160
0	100 ± 4.5	94.8 ± 5.0	86.9 ± 5.7	76.4 ± 6.0	42.8 ± 4.8	29.9 ± 5.4	17.7 ± 5.3
5	92.7 ± 4.1	83.7 ± 1.6* (CI = 0.73)	70.1 ± 2.3* (CI = 0.72)	70.5 ± 3.3 (CI = 1.19)	37.9 ± 3.4* (CI = 0.83)	31.3 ± 3.3 (CI = 1.12)	17.7 ± 3.7 (CI = 1.45)
10	82.5 ± 6.0	69.9 ± 6.0* (CI = 0.73)	49.3 ± 6.6* (CI = 0.51)	46.0 ± 3.1* (CI = 0.76)	32.0 ± 2.9* (CI = 0.66)	32.5 ± 5.1 (CI = 1.19)	22.1 ± 6.9 (CI = 1.49)
20	73.5 ± 6.0	49.0 ± 7.8* (CI = 0.64)	30.4 ± 9.7* (CI = 0.40)	33.2 ± 10.3* (CI = 0.53)	27.3 ± 2.0* (CI = 0.80)	25.9 ± 3.4 (CI = 1.32)	17.9 ± 4.5 (CI = 1.58)
40	48.5 ± 2.4	38.8 ± 2.1* (CI = 0.85)	26.9 ± 1.7* (CI = 0.67)	21.6 ± 1.9* (CI = 0.53)	20.6 ± 1.7* (CI = 0.71)	18.5 ± 2.4Δ (CI = 1.06)	18.3 ± 2.5 (CI = 1.76)
80	27.0 ± 1.9	24.6 ± 1.9 (CI = 1.15)	17.3 ± 1.6 (CI = 1.21)	17.8 ± 0.6* (CI = 0.89)	18.3 ± 0.7Δ (CI = 1.07)	13.9 ± 1.8 (CI = 1.42)	11.2 ± 1.5 (CI = 1.15)

Supplemental information on extraction, isolation and synthesis of YZL-51N, related to Figure 3.

Extraction and isolation of YZL-51N

The crude *P. americana* (30 kg) extract was prepared by reflux with 70% EtOH (3 × 120 L × 2 h) and suspended in water for extraction with EtOAc to obtain an EtOAc soluble extract (230 g). This extract was divided into six fractions (Fr.1–Fr.6) using a MCI gel CHP 20P column eluted with a gradient of aqueous MeOH (10%–100%). Fr. D (30 g) was further divided into four fractions (D1–D4) by Sephadex LH-20 (MeOH), of which, Fr. D2 (10 g) was again divided into eight fractions (D2.1–D2.8) by MCI gel CHP 20P eluted with gradient aqueous MeOH (10%–70%). Using an RP-18 column (MeOH/H_2O, 15%–50%), Fr. D2.3 (1.5 g) was divided into six portions (D2.3.1–D2.3.6). Fr. D2.3.5 (150 mg) was further purified by semi-preparative HPLC (aqueous acetonitrile, 15%) to yield **YZL-51N** (1.5 mg, t_R = 15.5 min).

YZL-51N: Yellow gum. ^{1}H NMR (600 MHz, CD_3OD) δ 7.54 (dd, *J* = 8.3, 2.2, H-6, 1H), 7.46 (d, *J* = 2.2, H-2, 1H), 7.38 (s, H-8, 1H), 7.05 (d, *J* = 2.3, H-2′, 1H), 7.01 (dd, *J* = 8.4, 2.3, H-6′, 1H), 6.87 (d, *J* = 8.3, H-5, 1H), 6.83 (d, *J* = 8.4, H-5′, 1H), 3.90 (s, OCH_3, 3H); ^{13}C NMR (150 MHz, CD_3OD) δ 188.9 (s, C-7), 172.1 (s, C-8′), 152.7 (s, C-3), 149.8 (s, C-7′), 149.0 (s, C-4′), 146.9 (s, C-3′), 146.8 (s, C-4), 131.2 (s, C-1), 126.8 (s, C-1′), 123.6 (d, C-6), 120.9 (d, C-6′), 118.9 (d, C-8), 116.7 (d, C-5′), 116.1 (d, C-2), 116.0 (d, C-5), 114.9 (d, C-2′), 53.0 (OCH_3). ESIMS: *m/z* 329 $[M - H]^-$; HRESIMS *m/z*: $[M - H]^-$ Calculated for $C_{17}H_{14}O_7$, 329,0667; Found 329.0664.

Elucidation of YZL-51N structure

YZL-51N (**Periplanol F)**, obtained as a yellowish gum, has the molecular formula $C_{17}H_{14}O_7$ as deduced from its HRESIMS, ^{13}C NMR, and DEPT spectra (11 degrees of unsaturation). The ^{1}H NMR spectrum of **YZL-51N** shows two ABX spin systems [δ_H 7.54 (dd, *J* = 8.3, 2.2, H-6, 1H), δ_H 7.46 (d, *J* = 2.2, H-2, 1H), δ_H 6.87 (d, *J* = 8.3, H-5, 1H); δ_H 7.05 (d, *J* = 2.3, H-2′, 1H), δ_H 7.01 (dd, *J* = 8.4, 2.3, H-6′, 1H), δ_H 6.83 (d, *J* = 8.4, H-5′, 1H)], an olefinic signal (δ_H, 7.38, s, H-7), and an methoxyl signal (δ_H 3.90, s, H-OCH_3). The ^{13}C NMR and DEPT spectra show 17 signals attributed to seven olefinic methine, and nine non-protonated carbons (1 ketone, 1 carbonyls, and 7 olefinic, including four oxygenated). The structure of **YZL-51N** was determined mainly based on 2D NMR spectroscopic data (Figure S13). The HMBC correlations of H-8/C-7, C-1′, C-7′, C-8′ and H-2′, H-6′/C-7′ suggested that C-7′ is linked to C-1′ and the presence of C-7-C-8-C-7′-C-8′ chain. In addition, the HMBC correlations of H-8/C-7 and H-2, H-6/C-7 indicated that C-7 is linked to C-1. The methyl esters were identified by the HMBC correlation between 8′-OCH_3 and C-8′. Further, the double bond configuration of **YZL-51N** was deduced as *Z* based on ROESY correlations of H-8/H-2′, H-6′. Thus, the structure of **YZL-51N** was finally assigned (Figure S12) and named periplanol F.

Synthesis of YZL-51N

YZL-51N was synthesized in a seven-step reaction with a total yield of 4% from the simple natural product derivatives 3,4-dimethoxyacetophenone and veratraldehyde. The detailed reaction processes are as follows in Scheme 1:

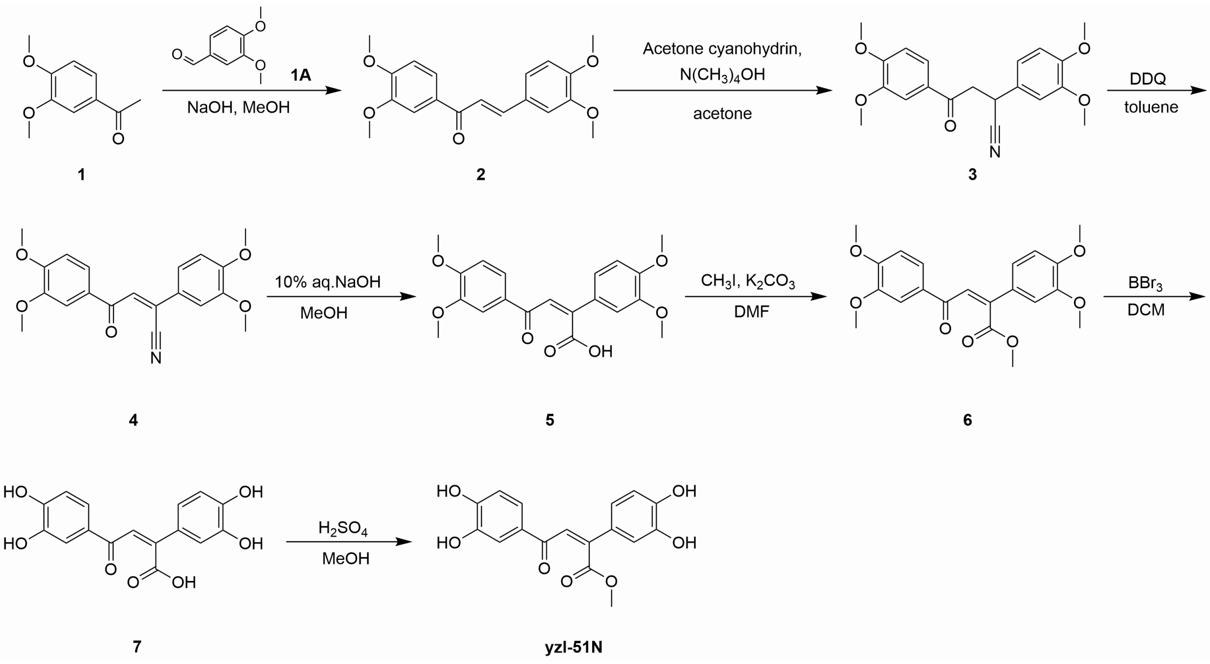

Scheme S1. Synthesis of YZL-51N, related to Figure 3.

General procedure for preparation of compound 2

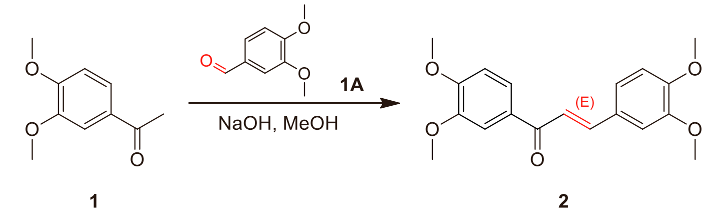

To a mixture of compounds **1** (450 g, 2.50 mol, 1.0 *eq*) and **1A** (435 g, 2.62 mol, 1.05 *eq*) in MeOH (2250 mL) was added NaOH (15.0 g, 374 mmol, 0.15 *eq*) in one portion at 25°C under Ar. The mixture was stirred at 60°C for 12 h. TLC (petroleum ether/EtOAc = 3/1, R_f = 0.20) showed the product. The mixture was cooled to 5°C and filtered in vacuum. The solid was washed with MeOH (1500 mL) at 0-10°C to obtain compound **2** (730 g, 2.22 mol, 89.0% yield) as a white solid. ^{1}H NMR (400 MHz, $CDCl_3$): δ 7.75–7.79 (m, 1H), 7.68–7.70 (m, 1H), 7.63 (s, 1H), 7.40–7.44 (m, 1H), 7.17 (s, 1H), 7.16 (s, 1H), 6.90–6.95 (m, 2H), 3.90–3.98 (m, 12H).

General procedure for preparation of compound 4

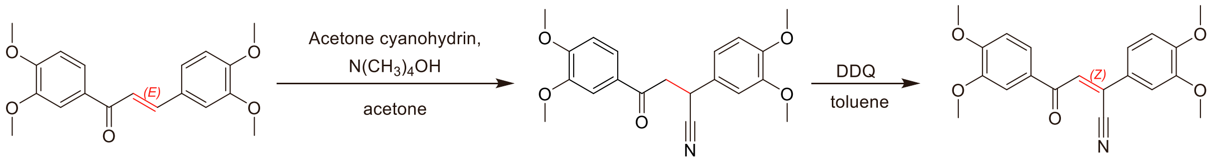

2 3 4

To a mixture of compound **2** (365 g, 1.11 mol, 1.0 *eq*) in acetone (1267 mL) and H_2O (34 mL) was added acetone cyanohydrin (118 g, 1.39 mol, 1.25 *eq*) and $N(CH_3)_4OH$ (12.1 g, 66.7 mmol, 0.06 *eq*) at 25°C. The mixture was stirred at 60°C for 10 h under N_2. TLC (petroleum ether/EtOAc = 3/1, R_f = 0.35) showed the product. The mixture was poured into ice-water (1200 mL) and stirred for 5 min. The mixture was filtered and the solid was concentrated in vacuum at 50°C to obtain compound **3** (720 g, 2.03 mol, 91.4% yield) as a light yellow solid. To a mixture of compound **3** (81.2 g, 228 mmol, 1.0 *eq*) in toluene (2400 mL) was added DDQ (64.8 g, 285 mmol, 1.25 *eq*) in one portion at 25°C under N_2. The mixture was stirred at 120°C for 6 h. TLC (petroleum ether/EtOAc = 3/1, R_f = 0.25) showed the product. The mixture was filtered and washed with dichloromethane (2000 mL). The solid was purified by re-crystallization from dichloromethane (4000 mL) and MeOH (1000 mL) to give compound **4** (330 g, 934 mmol, 51.1% yield) as a yellow solid which was used to the next step directly. ^{1}H NMR (400 MHz $CDCl_3$): δ 7.79 (s, 1H), 7.65 (s, 1H), 7.60–7.63 (m, 1H), 7.42–7.43 (m, 1H), 7.26–7.27 (m, 1H), 6.91–6.95 (m, 2H), 3.93–3.97 (m, 12H).

General procedure for preparation of compound 6

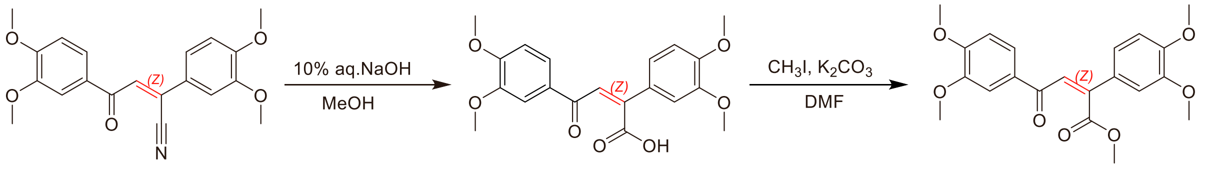

4 5 6

To a mixture of compound **4** (100 g, 283 mmol, 1.0 *eq*) and in H_2O (200 mL) and MeOH (500 mL) was added NaOH (250 g, 6.25 mol, 22 *eq*) in one portion at 25°C under N_2. The mixture was stirred at 60°C for 2 h. It was concentrated in vacuum. The residue was poured into H_2O (500 mL) and stirred for 5 min. The aqueous phase was adjusted to pH = 2.0 by 6N HCl and then extracted with EtOAc (800 mLx2). The combined organic phase was washed with brine (500 mL), dried with anhydrous Na_2SO_4, filtered and concentrated in vacuum to obtain compound **5** (135 g, crude) as a green solid. To a mixture of compound **5** (125 g, 335 mmol, 1.0 eq) in DMF (625 mL) was added K_2CO_3 (70.0 g, 503 mmol, 1.5 *eq*) and CH_3I (52.4 g, 369 mmol, 1.1 *eq*) in one portion at 20°C under N_2. The mixture was stirred at 20°C for 6 h. TLC (petroleum ether/EtOAc = 0/1, R_f = 0.45) showed the product. The mixture was filtered and the filtrate was poured into H_2O (2000 mL) and stirred for 5 min. The aqueous phase was extracted with EtOAc (2000 mLx2). The combined organic phase was washed with brine (1000 mL), dried with anhydrous Na_2SO_4, filtered and concentrated in vacuum, which was purified by silica gel

chromatography (petroleum ether/EtOAc = 10/1~ 0/1) to afford compound **6** (70.0 g, 163 mmol, 48.6% yield, 90% purity) as a yellow solid. ^{1}H NMR (400 MHz $CDCl_3$): δ 7.65–7.67 (m, 1H), 7.60–7.61 (m, 1H), 7.28 (s, 1H), 7.09–7.17 (m, 2H), 6.90–6.93 (m, 2H), 3.89–3.97 (m, 15H).

General procedure for preparation of YZL-51N

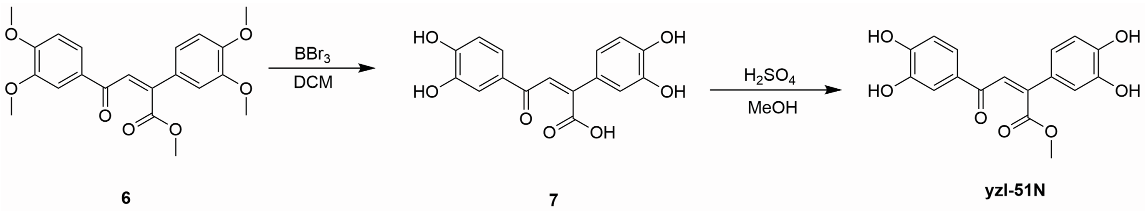

A mixture of compound **6** (35.0 g, 90.6 mmol, 1.0 *eq*) in dichloromethane (100 mL) was added to BBr_3 (908 g, 3.62 mol, 40 *eq*) dropwise at -40°C. The mixture was stirred at 0°C for 4 h. The mixture was poured into ice-water (w/w = 1/1) (3000 mL) and stirred for 5 min. The aqueous phase was extracted with EtOAc (1500mL). The organic phase was washed with brine (500 mL), dried with anhydrous Na_2SO_4, filtered and concentrated in vacuum to obtain compound **7** (55.0 g, crude) as a black oil which was used to the next step directly. To a mixture of compound **7** (42.0 g, 132 mmol, 1.0 *eq*) in MeOH (65 mL) was added H_2SO_4 (1.30 g, 13.0 mmol, 0.1 *eq*) in one portion at 25°C. The mixture was stirred at 60°C for 2 h. TLC (dichloromethane/methanol = 10/1, R_f = 0.20) showed the product. The mixture was concentrated in vacuum. The residue was poured into water (500 mL). The aqueous phase was extracted with EtOAc (250 mLx2). The combined organic phase was washed with brine (100 mL), dried with anhydrous Na_2SO_4, filtered and concentrated in vacuum. It was purified by silica gel chromatography (petroleum ether/EtOAc = 20/1~0/1), which was further purified by prep-HPLC: (column: Phenomenex luna C18 250*50mm*10 µm; mobile phase: [water (0.1%TFA)-ACN]; B%: 3%-33%, 20 min) to obtain **YZL-51N** (5.00 g, 15.0 mmol, 11.3% yield, 99.0% purity) as a yellow solid. ^{1}H NMR (500 MHz, CD_3OD): δ 7.54 (dd, J = 8.3, 2.2 Hz, 1H), 7.46 (d, J = 2.2 Hz, 1H), 7.38 (s, 1H), 7.06 (d, J = 2.3 Hz, 1H), 7.01 (dd, J = 8.3, 2.3 Hz, 1H), 6.87 (d, J = 8.3 Hz, 1H), 6.83 (d, J = 8.3 Hz, 1H), 3.90 (s, 3H); ^{13}C NMR (100 MHz, CD_3OD): δ 188.9, 172.1, 152.6, 149.8, 149.0, 146.9, 146.8, 131.2, 126.8, 123.6, 120.9, 118.9, 116.7, 116.1, 116.0, 114.9, 53.0. ESIMS: m/z 331 $[M + H]^+$.
